# EV-A71 induced IL-1β production in THP-1 macrophages is dependent on NLRP3, RIG-I, and TLR3

**DOI:** 10.1038/s41598-022-25458-x

**Published:** 2022-12-11

**Authors:** Hsing-I Huang, Chi-Chong Chio, Jhao-Yin Lin, Chia-Jung Chou, Chia-Chen Lin, Shih-Hsiang Chen, Liang-Sheng Yu

**Affiliations:** 1 Research Center for Emerging Viral Infections, College of Medicine, Chang Gung University, Kwei-Shan, Tao-Yuan, Taiwan; 2 Department of Medical Biotechnology and Laboratory Science, College of Medicine, Chang Gung University, Kwei-Shan, Tao-Yuan, Taiwan; 3 Graduate Institute of Biomedical Sciences, College of Medicine, Chang Gung University, Kwei-Shan, Tao-Yuan, Taiwan; 4 Department of Pediatrics, Linkou Chang Gung Memorial Hospital, Kwei-Shan, Tao-Yuan, Taiwan; 5 Division of Pediatric Hematology/Oncology, Linkou Chang Gung Memorial Hospital, Kwei-Shan, Tao-Yuan, Taiwan; 6 College of Medicine, Chang Gung University, Kwei-Shan, Tao-Yuan, Taiwan

**Keywords:** Cell biology, Immunology, Microbiology, Pathogenesis

## Abstract

Enterovirus A71 (EV-A71) is an emerging enterovirus that can cause neurological complications. Enhanced serum IL-1β levels were observed in EV-A71 patients with severe neurological symptoms. However, the roles of sensors in enterovirus-induced IL-1β production are unclear. In this study, we identified that pattern recognition receptors, including RIG-I, TLR3, and TLR8, are implicated in EV-A71-triggered IL-1β release in human macrophages. EV-A71 infection results in caspase-1 and caspase-8, which act as regulators of EV-A71-induced NLRP3 and RIG-I inflammasome activation. Moreover, knockdown of the expression of TLR3 and TLR8 decreased the released IL-1β in an NLRP3-dependent manner. Since TLR3 and TLR8 ligands promote NLRP3 inflammasome activation via caspase-8, the alternative pathway may be involved. In summary, these results indicate that activation of the NLRP3 and RIG-I inflammasomes in EV-A71-infected macrophages is mediated by caspase-1 and caspase-8 and affected by TLRs, including TLR3 and TLR8.

## Introduction

EV-A71 is a positive-sense RNA virus that belongs to the family Picornaviridae. EV-A71 infection is associated with several diseases, including hand, foot, and mouth disease (HFMD) and herpangina. Moreover, EV-A71 may invade the central nervous system and cause severe neurological complications, causing significant concern in the Asia–Pacific region^1^. The severity of EV-A71 infection has been suggested to be correlated with the production of proinflammatory cytokines^2^. Previous studies indicated that the production of inflammatory cytokines such as IL-6 and IL-1β might be involved in central nervous system (CNS) damage caused by EV-A71 infection^3,4^.

Previous studies have demonstrated that macrophages are attributed to IL-1β production during viral infections. As the primary effector cells of the innate immune system, macrophages play essential roles in the recognition and destruction of invading microorganisms. When exposed to pathogens or inflammatory stimuli, they release cytokines and chemokines to induce enhanced vascular permeability and recruitment of immune cells^5^. However, many viruses can infect and replicate in macrophages to facilitate their dissemination^6^. Macrophage infection induces the expression of cytokines, which is associated with disease severity^7^.

Moreover, emerging evidence suggests that viral infection can activate inflammasomes in macrophages to produce IL-1β and IL-18^8,9^. Excessive IL-1β expression is associated with virus pathogenesis. For instance, Theiler's murine encephalomyelitis virus (TMEV) induced demyelinating disease by promoting the generation of Th17 cells^10^. Furthermore, a recent study indicated that the nucleotide-binding oligomerization domain 3 (NLRP3) inflammasome is involved in neuroinvasion and neuroinflammation during severe acute respiratory syndrome coronavirus-2 (SARS-CoV-2) infection^11^.

The production of IL-1β depends on inflammasome activation, which are composed of multiple proteins, including NOD-like receptors (NLRs), the adaptor protein apoptosis-associated speck-like protein containing caspase recruitment domain (CARD) known as ASC, and the effector protein pro-caspase-1. Recently, several types of inflammasomes were characterized. Accumulated studies have revealed that NLRP1, NLRP3, NLR family CARD domain-containing 4 (NLRC4) and pyrin inflammasome activation are mediated by NLR family members, while the absent in melanoma 2 (AIM2) inflammasome is activated by AIM2, which belongs to the Ifi202/IFI116 family^12,13^. RNA viruses such as influenza A virus (IAV), human immunodeficiency virus (HIV), and encephalomyocarditis virus (EMCV) can induce NLRP3 inflammasome activation and the subsequent generation of IL-1β^14–16^.

The formation of NLRP3 inflammasomes has been demonstrated to be involved in viral pathogenesis^11,17,18^. The NLRP3 inflammasome can be activated by various stimuli, such as extracellular ATP, asbestos, silica, alum, amyloid-β, single-stranded RNA (ssRNA), double-stranded (dsRNA) analogs, and pathogen products^19^. Accumulating evidence has demonstrated that picornaviruses, including EMCV, coxsackievirus B3 (CVB3), and poliovirus (PV), can produce IL-1β^16,20^. The induction of IL-1β is implicated in CVB3-induced myocarditis, and the transplantation of NLRP3-knockdown (KD) macrophages results in reduced IL-1β secretion and milder symptoms^20^, indicating that IL-1β plays an essential role in pathogenesis. In human rhinovirus (HRV)-infected airway epithelial cells, inflammasome-dependent IL-1β secretion exacerbates pulmonary symptoms^21^.

Interestingly, the production of IL-1β can result in the pyroptosis of EV-A71-infected neural cells, which suggests that inflammasome activation may be involved in programmed cell death^3^. In contrast, the production of IL-1β has been reported to play beneficial roles during enteroviral infections. For example, Wang et al. showed that CVB3 infection resulted in more severe symptoms in NLRP3-knockout mice, while the NLRP3 inflammasome was demonstrated to elicit beneficial effects on EV-A71-infected animals^22,23^. The conflicting results of these studies suggest that inflammasome activation may exacerbate or ameliorate disease severity during enteroviral infections.

Previous research has shown that the NLRP3 inflammasome is involved in EV-triggered IL-1β release^23,24^. However, the mechanisms associated with EV-A71-induced IL-1β production in macrophages have not been thoroughly characterized. In this study, we showed that caspase-1 and caspase-8 are involved in IL-1β production and that both NLRP3 and RIG-I inflammasomes are formed in EV-A71-infected human macrophages. Moreover, the expression levels of TLR3 and TLR8 influenced inflammasome activation, indicating that RNA sensors are involved. We also showed that EV-A71 viral RNAs are sufficient to trigger IL-1β release in macrophages in an NLRP3- and RIG-I-dependent manner. Taken together, our study provides evidence of the roles of TLRs, RIG-I, and caspase-8 in enterovirus-induced inflammasome activation.

## Results

### Caspase-8 is involved in EV-A71-induced inflammasome activation in human macrophages

At first, the permissiveness and ability to induce IL-1β production were examined in human monocytes and macrophages using THP-1 and PMA-primed THP-1 cells as cellular models. Our results showed that both cell types are permissive to EV-A71 infection (Supplementary Fig. 1A–C). The production of IL-1β transcripts and proteins was significantly increased in PMA-primed THP-1 compared to untreated cells (Supplementary Fig. 1C and D). Furthermore, increased IL-1β release was also observed in human peripheral blood-derived monocytes (PBMCs)-differentiated macrophages upon EV-A71 infection (Supplementary Fig. 1E–G). This result is in concordance with the previous report^25^.

**Figure 1:**
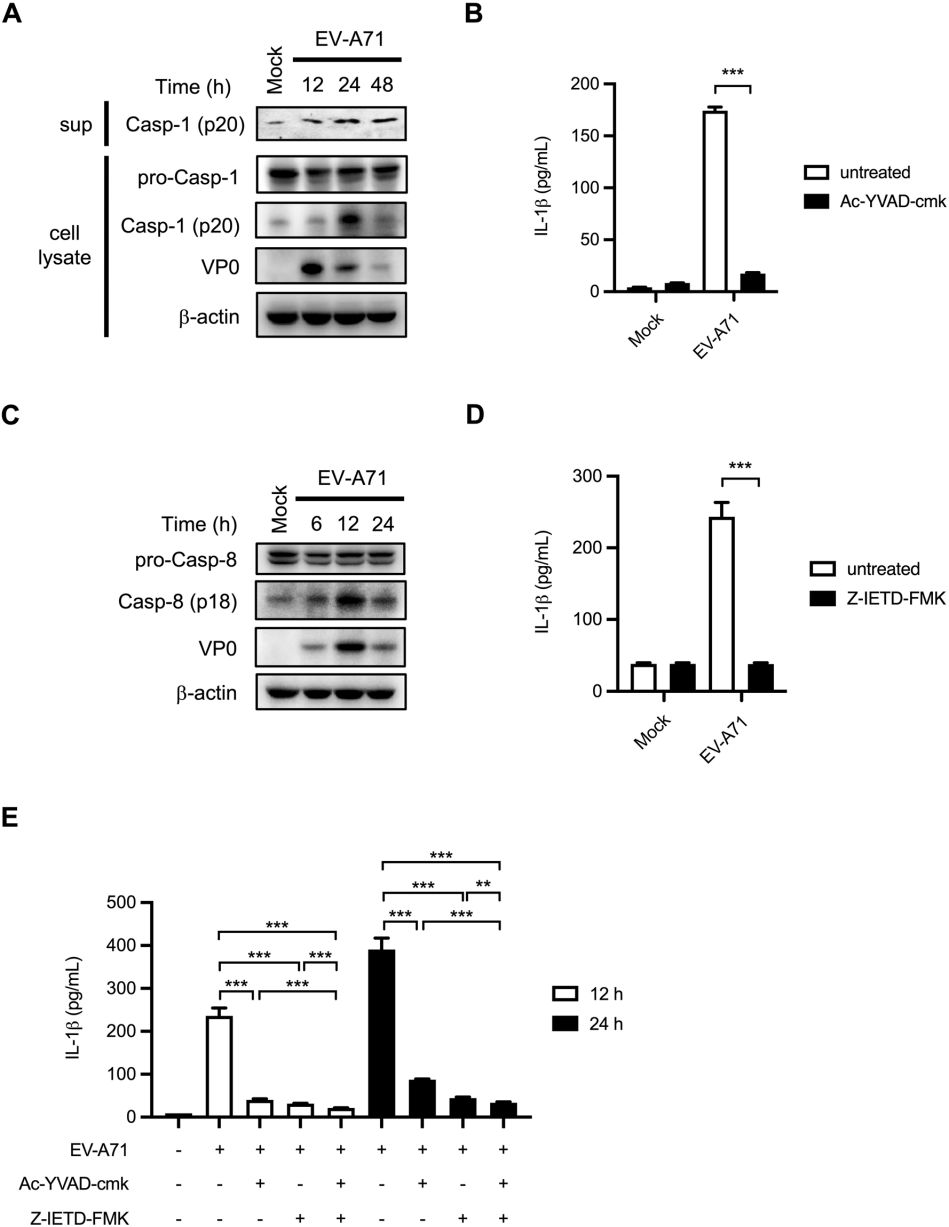
EV-A71 infection induced IL-1β secretion is dependent on caspase-1 and caspase-8. (**A**) PMA-primed THP-1 cells were infected with EV-A71 (2 M.O.I.). The expression levels of cleaved caspase-1, pro-caspase-1 and EV-A71 viral protein VP0 was analyzed by Western blot. β-actin was used as a loading control. (**B**) THP-1 macrophages were treated with the caspase-1 inhibitor (Ac-YVAD-cmk) at the concentration of 1 μM. The cells were then infected with EV-A71 at the M.O.I. of 2 for 12 h. IL-1β levels in the supernatant were determined by ELISA. (**C**) PMA-primed THP-1 cells were infected with EV-A71 at an M.O.I. of 2. Cell lysates were harvested at 6, 12, and 24 h post-infection. Pro-caspase-8, cleaved caspase-8, EV-A71 VP0, and β-actin were determined by Western blot. (**D**) THP-1 macrophages were treated with the caspase-8 inhibitor (Z-IETD-FMK) at the concentration of 20 μM and infected with EV-A71 for 12 h. The secreted IL-1β levels were quantified by ELISA. (E) THP-1 macrophages were treated with Ac-YVAD-cmk, Z-IETD-FMK, or Ac-YVAD-cmk + Z-IETD-FMK after virus absorption. Mature IL-1β levels in the supernatants were determined by ELISA. Data are expressed as mean value ± SD (**p < 0.01, ***p < 0.001, Student’s unpaired T-test).

Many studies have implicated caspase-1 in virus infection-associated inflammasome activation^25–27^. Our results showed that EV-A71 infection increased the expression of cleaved caspase-1 in supernatants collected from infected THP-1 macrophages (Fig. 1A). Furthermore, treatment with Ac-YVAD-cmk, a caspase-1 inhibitor, resulted in more than 80% downregulation of released IL-1β in EV-A71-infected cells (Fig. 1B). A previous study showed that caspase-8 might act as a maturing enzyme to catalyze the production of IL-1β^28^. To determine whether caspase-8 is involved with EV-A71-induced IL-1β release in macrophages, we examined the activation of caspase-8 in virus-infected THP-1 macrophages. Our results revealed that the expression of cleaved caspase-8 was detected in THP-1 macrophages upon EV-A71 infection (Fig. 1C). To understand whether caspase-8 activation is implicated in EV-A71-induced IL-1β production, PMA-primed THP-1 cells were treated with Z-IETD-FMK, the caspase-8 inhibitor, and infected with EV-A71. Interestingly, the amounts of secreted IL-1β were drastically decreased in Z-IETD-FMK-treated cells (Fig. 1D). The cytotoxicity assay was performed to confirm that our observed differences did not come from cell death (Supplementary Fig. 2). Thus, our data showed that caspase-8 activation is essential for EV-A71-promoted inflammasome activation. Furthermore, treated cells with Z-IETD-FMK, Ac-YVAD-cmk, and the combination of Z-IETD-FMK and Ac-YVAD-cmk showed no significant synergistic effect (Fig. 1E), suggesting that caspase-1 and caspase-8 act as components on the same pathway.

### Activation of NLRP3 and RIG-I inflammasomes contributes to EV-A71-induced IL-1β production

**Figure 2:**
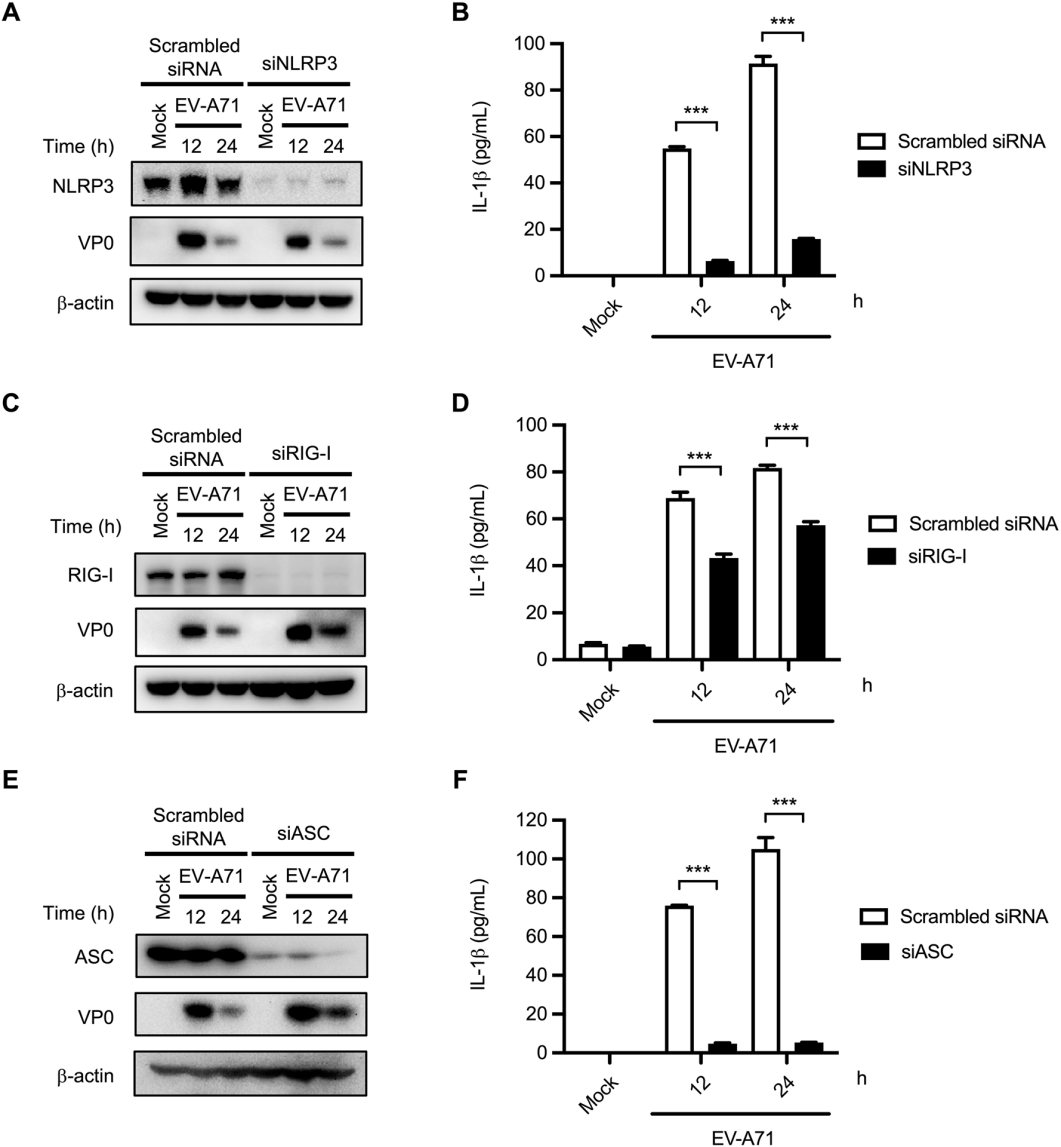
NLRP3 and RIG-I are involved with EV-A71 -triggered IL-1β production in THP-1 macrophage. (**A**, **B**) NLRP3 siRNA (siNLRP3) (100 nM) or scrambled siRNA was transfected into PMA-primed THP-1 cells for 48 h. Then the cells were infected with EV-A71 at an M.O.I. of 2 for 12 h. (**A**) NLRP3, EV-A71 VP0, and β-actin were analyzed by Western blot. (**B**) The secretion levels of IL-1β were assessed by ELISA. (**C**, **D**) PMA-primed THP-1 cells were transfected 100 nM siRNA target RIG-I (siRIG-I) or scrambled siRNA for 48 h, and then infected with 2 M.O.I. EV-A71 for 24 h. (**C**) The expression levels of RIG-I, EV-A71 VP0, and β-actin were determined by Western blot. (**D**) Released IL-1β levels were quantified by ELISA. (**E**, **F**) Specific ASC siRNA (siASC) and scrambled siRNA were transfected into PMA-primed THP-1 cells for 48 h. Cells were then infected with EV-A71 at an M.O.I. of 2 for 12 h. (**E**) The expression levels of ASC, EV-A71 VP0, and β-actin were analyzed by Western blot. (**F**) The supernatants were collected to quantify the released IL-1β protein levels by ELISA. Data are expressed as mean value $\pm$ SD (***p < 0.001, Student’s unpaired T-test).

Various RNA viruses, including HCV, CVB3, and EV-A71, have been demonstrated to activate multiple types of inflammasomes for IL-1β production^20,29,30,32^. To examine whether NLRP3 is involved in EV-A71-induced inflammasome formation in macrophages, PMA-primed THP-1 cells were transfected with scrambled siRNA or NLRP3 siRNA and then infected with EV-A71. Western blot analysis was performed to confirm the knockdown efficiency of NLRP3 at the protein level (Fig. 2A). The levels of secreted IL-1β protein were quantified by ELISA. As shown in Fig. 2B, IL-1β production was decreased in NLRP3-KD cells compared to scrambled siRNA-transfected THP-1 macrophages. These results indicated that EV-A71 activates the NLRP3 inflammasome in human mononuclear phagocytes. The results are similar to those of previously published research^23,24^. In addition, to examine whether RIG-I is involved in IL-1β production in EV-A71-infected macrophages, the specific siRNA target RIG-I was used to suppress RIG-I protein expression. The knockdown efficiency was confirmed by immunoblot (Fig. 2C). The production of mature IL-1β in EV-A71-infected macrophages was significantly reduced in RIG-I-KD cells compared to those transfected with scramble siRNA (Fig. 2D). Together, these data suggested that NLRP3 and RIG-I inflammasome were involved in EV-A71-induced IL-1β production. To examine the role of ASC in EV-A71-induced IL-1β production, siASCs were transfected into THP-1 macrophages, and EV-A71-triggered IL-1β protein release was examined in ASC knockdown and control cells. Immunoblotting was performed to confirm the knockdown efficiency and viral infection (Fig. 2E). ELISA results revealed that EV-A71-induced IL-1β expression was abolished in ASC knockdown cells (Fig. 2F).

### EV-A71-induced RIG-I inflammasome activation is independent of NLRP3

**Figure 3:**
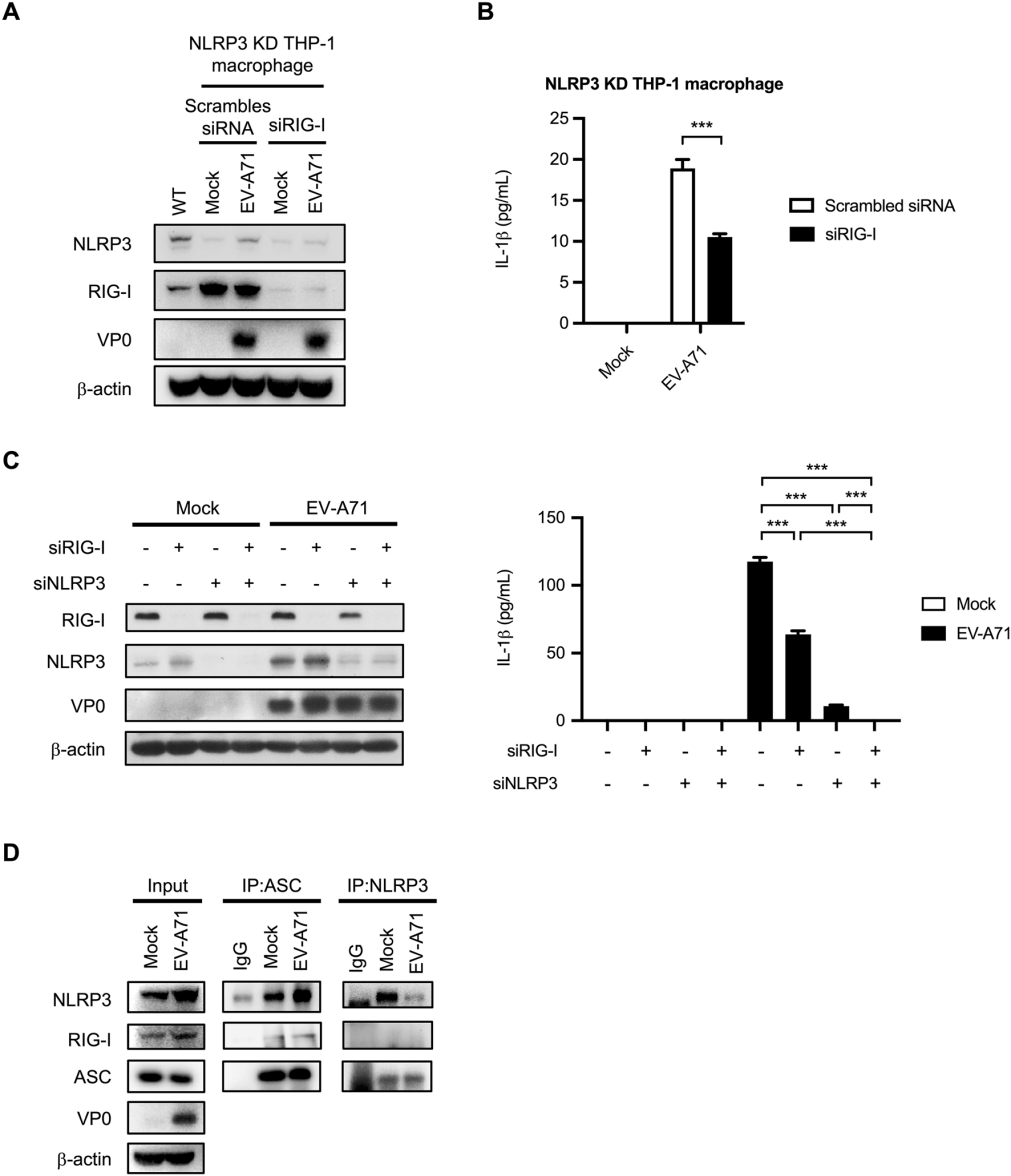
RIG-I contributes to IL-1β production in EV-A71-infected macrophages in an NLRP3-independent manner. (**A**) The NLRP3 KD THP-1 cells were primed with PMA for 48 h for differentiation. The primed cells were subsequently transfected with siRIG-I and scrambled siRNA for 48 h and then infected with EV-A71 (M.O.I. = 2). The expression levels of NLRP3, RIG-I, EV-A71 VP0, and β-actin were examined by Western blot. (**B**) The supernatants were harvested at 12 h p.i.. The IL-1β levels were determined by ELISA. (**C**) THP-1 macrophages were transfected with siNLRP3, siRIG-I, and siNLRP3 + siRIG-I for 48 h, respectively. The transfected cells were subsequently infected with EV-A71 at the M.O.I. of 2. The expression levels of RIG-I, NLRP3, EV-A71 VP0, and β-actin were examined by Western blot. IL-1β levels in the supernatants were determined by ELISA. (**D**) Lysates prepared from mock- or EV-A71-infected PMA-primed THP-1 cells were subjected to immunoprecipitation with anti-ASC or anti-NLRP3 antibodies attached to sepharose. Whole lysates and immunoprecipitates were analyzed by immunoblotting with anti-NLRP3, anti-RIG-I, and anti-ASC. The expression of β-actin was used as an internal control. Data are expressed as mean value $\pm$ SD (***p < 0.001, Student’s unpaired T-test).

To further investigate whether RIG-I can affect EV-A71-induced IL-1β release in an NLRP3-independent manner, NLRP3 KD THP-1 macrophages were transfected with siRIG-I and then infected with EV-A71. The expression levels of RIG-I, NLRP3, and EV-A71 VP0 were confirmed by Western blotting (Fig. 3A). The supernatants were harvested at 12 h post-infection and subjected to ELISA to determine IL-1β protein expression levels. The data revealed that RIG-I knockdown decreased IL-1β production in EV-A71-infected NLRP3 KD THP-1 macrophages (Fig. 3B). Furthermore, a double knockdown experiment was performed to decipher the roles of RIG-I and NLRP3 in EV-A71-triggered IL-1β production. Our results revealed that RIG-I knockdown resulted in an approximately 50% decrease in IL-1β production, while NLRP3 KD led to an ~ 85% downregulation of IL-1β secretion. Interestingly, EV-A71 infection failed to induce IL-1β release in cells that lacked RIG-I and NLRP3 expression (Fig. 3C). Therefore, both NLRP3 and RIG-I can affect inflammasome activation in EV-A71-infected macrophages. Next, we sought to determine whether the RIG-I protein directly interacts with ASC, the major protein of the inflammasome. An immunoprecipitation assay was performed, and the results showed that the RIG-I protein was pulled down with ASC in EV-A71-infected cells in an NLRP3-independent manner (Fig. 3D).

### Caspase-1 and caspase-8 are implicated in RIG-I agonist-induced IL-1β production in THP-1 macrophages

**Figure 4:**
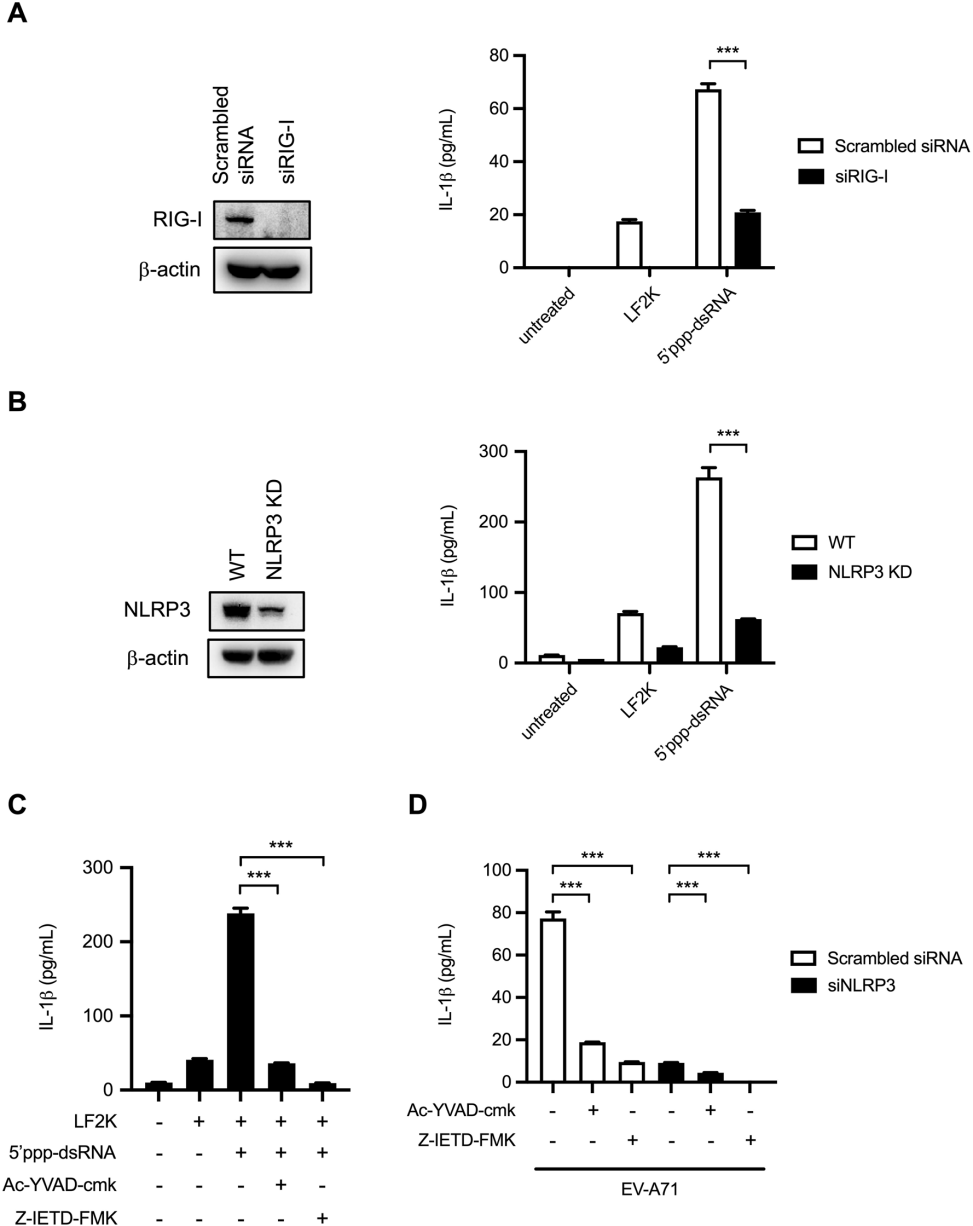
RIG-I agonist induced IL-1β secretion in dependent on NLRP3, caspase-1 and caspase-8. (**A**) PMA-primed THP-1 cells were transfected with siRIG-I (100 nM) or scrambled siRNA for 48 h prior to infection. Then the transfected cells were treated with RIG-I agonist 5’ppp-dsRNA for 12 h. The expression levels of RIG-I and β-actin were determined by Western blot. ELISA was applied to quantify the secreted IL-1β protein. (**B**) NLRP3 KD THP-1 cells were treated with 200 nM PMA for 48 h. Then the PMA-primed THP-1 cells were treated with 5’ppp-dsRNA for 12 h. The expression levels of NLRP3 and β-actin were determined by Western blot. The secreted IL-1β levels were determined by ELISA. (**C**) PMA-primed THP-1 cells were transfected with 5’ppp-dsRNA and then treated with Ac-YVAD-cmk or Z-IETD-FMK. The secreted IL-1β levels in the supernatant were determined by ELISA. (**D**) PMA-primed THP-1 cells were transfected with siNLRP3 or scrambled siRNA for 48 h prior to infection. The transfected cells were infected with EV-A71 and then treated with Ac-YVAD-cmk or Z-IETD-FMK. Supernatants were harvested at 12 h post-infection, and the secreted IL-1β levels were examined by ELISA. Data are expressed as mean value $\pm$ SD (***p < 0.001, Student’s unpaired T-test).

To further elucidate the role of RIG-I in inflammasome activation, the RIG-I agonist 5’ppp-dsRNA was transfected into THP-1 macrophages, and the expression of IL-1β protein was examined. The data showed that the amount of released IL-1β was increased by RIG-I engagement. Furthermore, transfection of RIG-I siRNA resulted in decreased RIG-I agonist-induced IL-1β production (Fig. 4A). To elucidate the effect of NLRP3 expression on RIG-I agonist-stimulated inflammasome activation, 5’ppp-dsRNA was transfected into NLRP3-KD THP-1 macrophages. Our results showed that knockdown NLRP3 decreased IL-1β production in RIG-I agonist-treated macrophagic cells, suggesting that NLRP3 could affect RIG-I inflammasome activation, even though these two proteins cannot interact with each other (Fig. 4B). To examine whether caspase-1 and caspase-8 are implicated in RIG-I inflammasome formation in macrophages, PMA-primed THP-1 cells were transfected with RIG-I agonist and then treated with caspase-1 or caspase-8 inhibitors. Our results showed that both caspase-1 and caspase-8 inhibitors could influence RIG-I engagement-induced IL-1β production, suggesting both caspases are implicated with RIG-I inflammasome activation (Fig. 4C). Furthermore, caspase-1 and caspase-8 are involved in EV-A71-induced RIG-I inflammasome activation since their inhibitors can significantly decrease the maturation of IL-1β in NLRP3-KD macrophages (Fig. 4D).

### TLR3 and TLR8 KD suppresses EV-A71-induced IL-1β production

**Figure 5:**
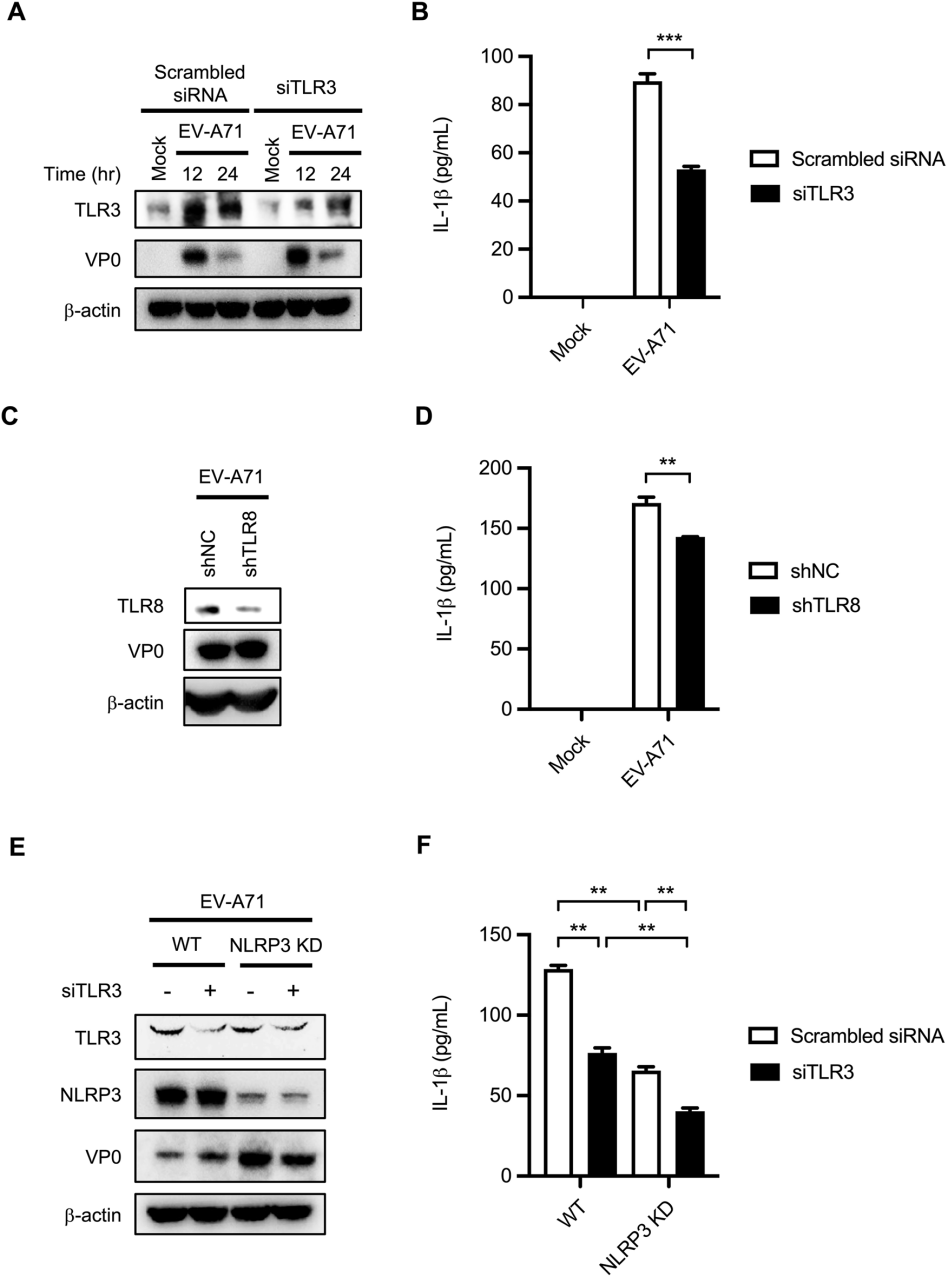
TLR3 and TLR8 are involved with EV-A71-induced IL-1β production in THP-1 macrophages. (**A**) Specific TLR3 siRNA or scrambled siRNA was transfected into PMA-primed THP-1 cells for 48 h. Cells were then infected with EV-A71 at the M.O.I. of 2 for 12 h. The expression of TLR3, EV-A71 viral protein VP0, and β-actin was analyzed by Western blot. (**B**) The IL-1β levels in the supernatants were determined by ELISA. (**C**) THP-1 cells were transduced with shNC or shTLR8. The transfected cells were selected and maintained in the puromycin-containing medium. The selected cells were infected with EV-A71 at the M.O.I. of 2 for 12 h. The expression levels of TLR8 and EV-A71 VP0 were examined by Western blot analysis. (**D**) The IL-1β levels in supernatants were assessed by ELISA. (**E**) Wildtype (WT) and NLRP3 KD THP-1 macrophages were transfected with siTLR3 or scrambled siRNA. The transfected cells were infected with EV-A71 at the M.O.I. of 2 for 12 h. The expression levels of TLR3, NLRP3, EV-A71 VP0, and β-actin were determined by Western blot analysis. (**F**) The supernatant was collected and the secreted IL-1β was analyzed by ELISA. Data are expressed as mean value $\pm$ SD (**p < 0.01, ***p < 0.001, Student’s unpaired T-test).

TLRs are known to be implicated in pathogen-induced IL-1β production^31^. Among these TLRs, evidence suggests that TLR3 and TLR8 play roles in RNA virus-triggered IL-1β maturation^14,32,33^. To investigate which TLRs are involved in EV-A71-induced IL-1β production in human macrophages, siRNAs specific to TLR3 were transfected into THP-1 macrophages, subsequently infected with EV-A71. The knockdown efficiency was examined by Western blot analysis (Fig. 5A). The expression of proteins was measured by ELISA, which showed that the knockdown of TLR3 resulted in decreased IL-1β secretion (Fig. 5B). Lentiviral vectors carrying TLR8 shRNAs were transduced into THP-1 cells to select stable TLR8-KD clones. TLR8-KD THP-1 cells were treated with PMA to force their macrophagic differentiation and then infected with EV-A71. The expression levels of TLR8 and the viral protein VP0 were analyzed by Western blotting (Fig. 5C). The secreted mature IL-1β protein was examined by ELISA, which showed that the expression levels of secreted proteins were downregulated in TLR8-KD cells (Fig. 5D). Furthermore, to investigate whether TLR3-promote IL-1β secretion is in an NLRP3-dependent manner, the wild type and NLRP3-KD THP-1 macrophages were transfected with TLR3 siRNA and then infected by EV-A71. The knockdown efficiency was confirmed by Western blot (Fig. 5E). Our results show that the suppressive effect of TLR3 knockdown on IL-1β release was less evident in NLRP3 KD cells (Fig. 5F).

### Engagement of TLR3 and TLR8 induces IL-1β maturation in THP-1 macrophages

**Figure 6:**
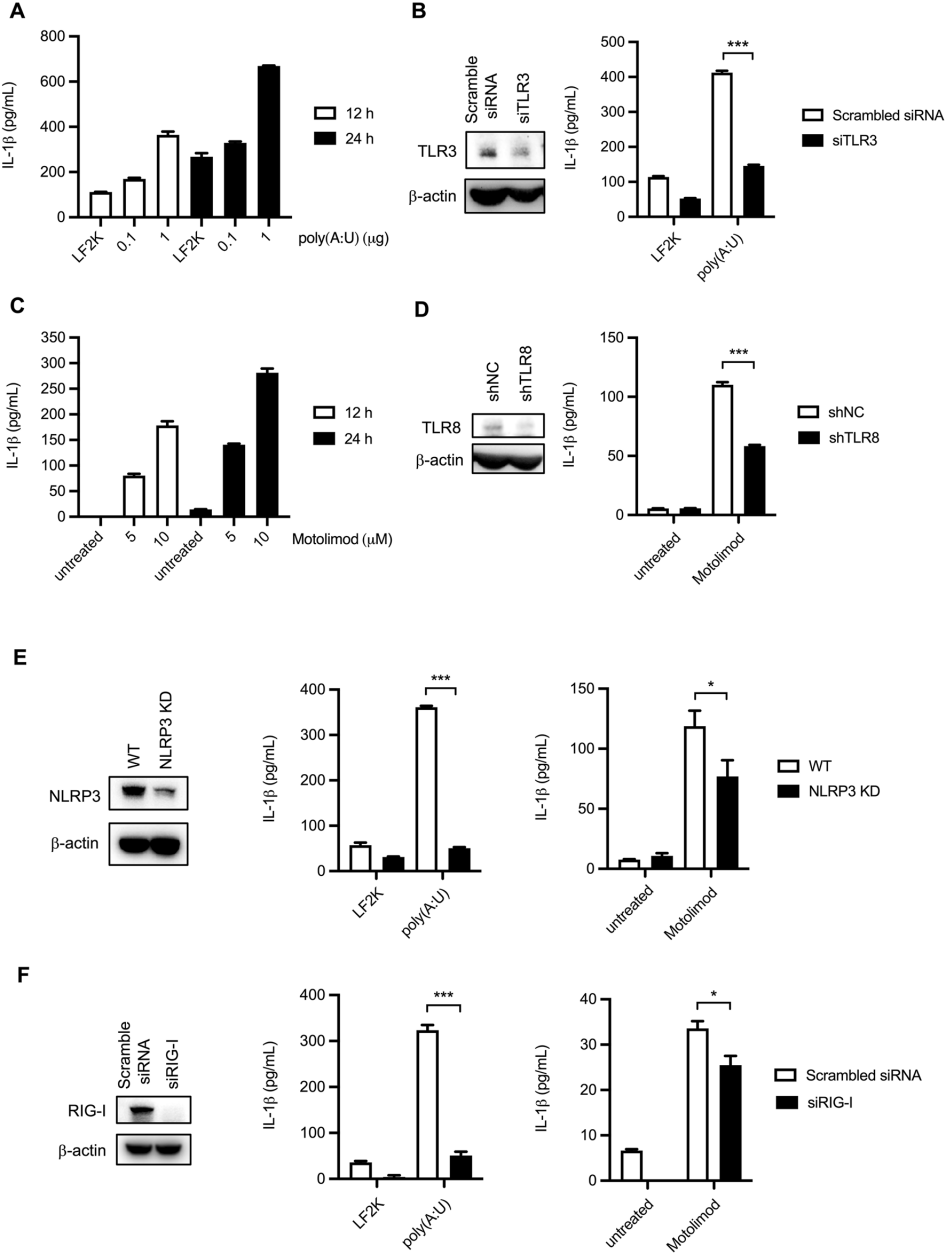
NLRP3 and RIG-I are implicated in TLR3 and TLR8 induced IL-1β release in THP-1 macrophages. (**A**) THP-1 macrophages were stimulated by poly(A:U) (0.1, 1 µg) for 12 and 24 h. The IL-1β protein levels were detected by ELISA. (**B**) PMA-primed THP-1 were transfected with TLR3 siRNA and scrambled siRNA for 48 h, and then stimulated by 1 µg poly(A:U) for 12 h. The knockdown efficiency was confirmed by Western blot. The expression levels of IL-1β in the supernatant were analyzed by ELISA. (**C**) PMA-primed THP-1 cells were treated with TLR8 agonists motolimod (5, 10 µM) for 12 and 24 h. The supernatant was collected and the expression of IL-1β was examined by ELISA. (**D**) The shTLR8 transduced THP-1 macrophages were treated with motolimod for 12 h. TLR8 and β-actin in the cell lysates were detected by Immunoblot. IL-1β levels were quantified by ELISA. (**E**) The NLRP3 KD THP-1 macrophage was transfected with poly(A:U), or treated with motolimod for 12 h. The IL-1β levels in the supernatants were examined by ELISA. (**F**) PMA-primed THP-1 was transfected with siRIG-I and scrambled siRNA for 48 h. Then the transfected cells were stimulated by poly(A:U) or motolimod for 12 h. The expression of RIG-I and β-actin was determined by Western blot. The secretion of IL-1β was measured by ELISA. Data are expressed as mean value $\pm$ SD (*p < 0.05, ***p < 0.001, Student’s unpaired T-test).

TLR engagement has been demonstrated to be sufficient to promote inflammasome activation in human monocytes. To test the effects of TLR3 and TLR8 engagement in triggering IL-1β production in THP-1 macrophages, we stimulated the cells with poly(A:U), the TLR3 agonist, or motolimod, the TLR8 agonist and analyzed IL-1β release by ELISA. Our results revealed that both poly(A:U) and motolimod-induced IL-1β were released from THP-1 macrophages. (Figs. 6A and C). To confirm the effects of TLR engagement, the expression of TLR3 and TLR8 was knocked down by transfecting siTLR3 and transduction of shTLR8 into cells, respectively. TLR3 and TLR8 ligands were then added to stimulate macrophages. Our results showed that TLR3 and TLR8 agonist-triggered IL-1β release was diminished in TLR knockdown cells (Figs. 6B and D). The cleaved caspase-8 could be detected in poly(A:U) transfected THP-1 macrophages (Supplementary Fig. 3).To examine the involvement of NLRP3 and RIG-I in TLR engagement-triggered IL-1β production, NLRP3-KD, and RIG-I-KD THP-1 macrophages were stimulated with TLR3 and TLR8 ligands, respectively. Our data showed that the IL-1β production triggered by TLR3 and TLR8 engagements was affected by the expression of NLRP3 and RIG-I. NLRP3 knockdown decreased 75% of TLR3 stimulation-induced IL-1β release while downregulating only approximately 25% of TLR8-triggered IL-1β production (Fig. 6E). In addition, the expression of RIG-I also influenced both TLR3- and TLR8-induced IL-1β release (Fig. 6F).

### Virus RNA induces IL-1β production by promoting caspase-8 activation

**Figure 7:**
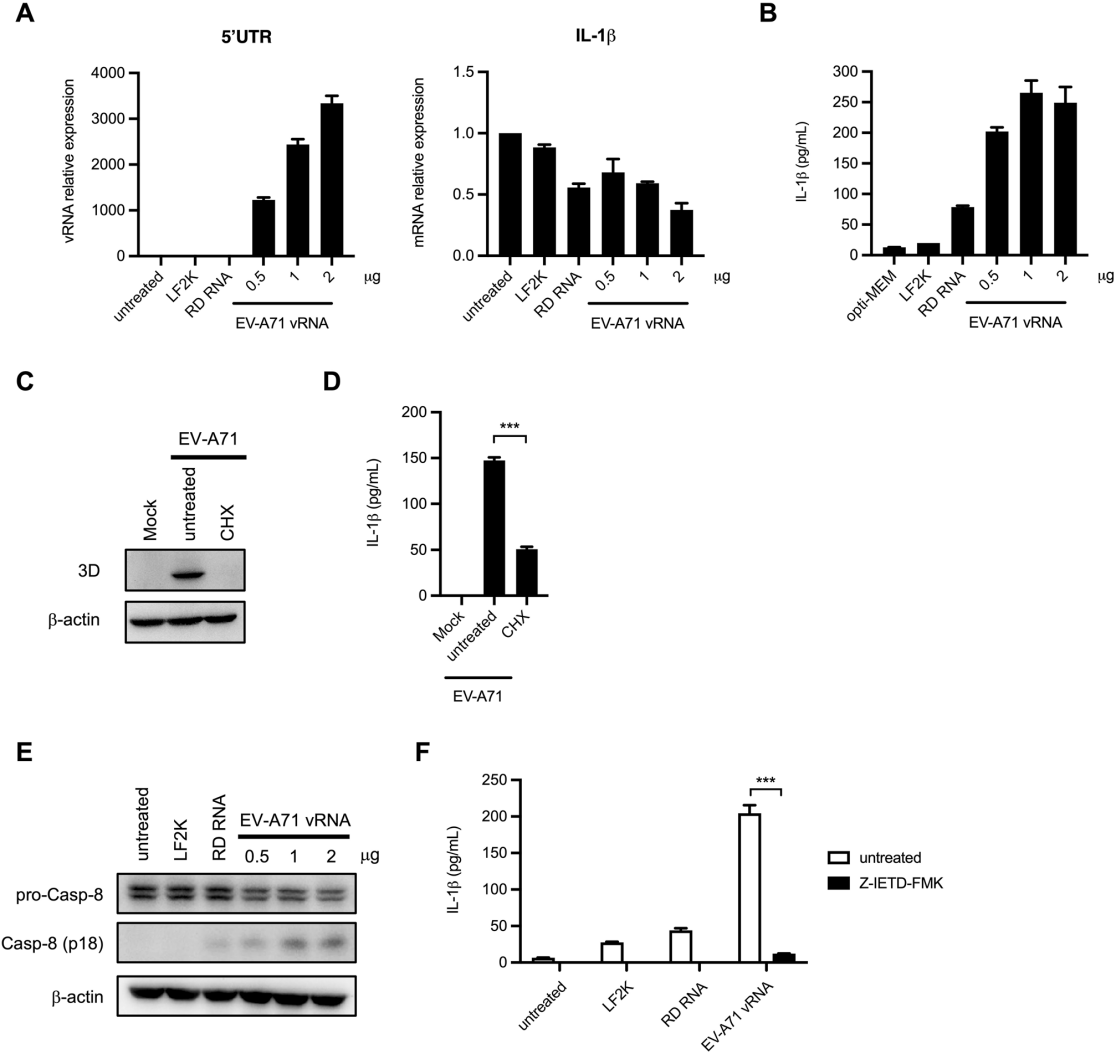
EV-A71 vRNA-triggered IL-1β release is dependent on active caspase-8. (**A**) Different amounts of EV-A71 viral RNA (0.5, 1 and 2 µg) and 1 µg RD cells RNA were transfected into PMA-primed THP-1 cells for 12 h. The expression levels of EV-A71 5’UTR and IL-1β transcripts were examined by RT-qPCR. (**B**) Supernatants were collected and the expression of IL-1β was analyzed by ELISA. (**C**) PMA-primed THP-1 cells were treated with cycloheximide (CHX) (100 μM) for 12 h to suppress the de novo protein synthesis. The untreated and CHX-treated THP-1 macrophages were infected with EV-A71 for 12 h. The expression of EV-A71 3D protein and β-actin was examined by Western blot. (**D**) The released IL-1β levels were determined by ELISA. (**E**) THP-1 macrophages were transfected with 1 µg RD cellular RNA or EV-A71 vRNA (0.5, 1, and 2 µg). The expression of pro-caspase-8, cleaved caspase-8, and β-actin was examined by immunoblot. (**F**) THP-1 macrophages were stimulated by 1 µg RD RNA or EV-A71 vRNA. Then the cells were treated with Z-IETD-FMK for 12 h. The supernatants were harvested and subjected to IL-1β ELISA. (***p < 0.001, Student’s unpaired T-test).

**Figure 8:**
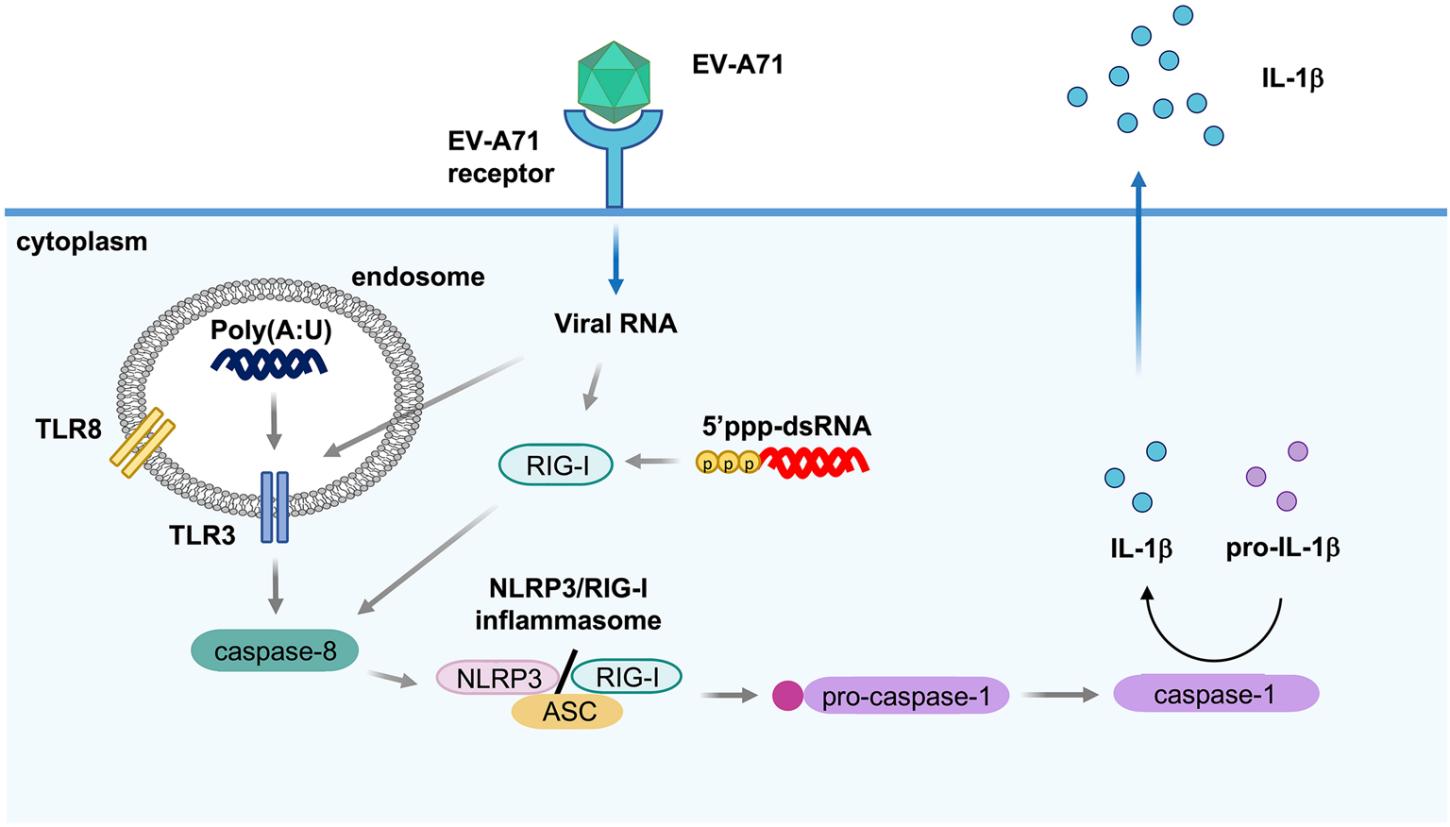
Mechanism of EV-A71 vRNA-triggered NLRP3-, RIG-I-inflammasome. Following the EV-A71 infection, TLR3 recognized EV-A71 vRNA and induced caspase-8 activation. The activation of caspase-8 promote the NLRP3-ASC and RIG-I-ASC assembly, and cleavage of caspase-1 cleavages pro-IL-1β to mature IL-1β.

To determine the potential of viral RNAs in triggering IL-1β release, different amounts of EV-A71 vRNA were transfected into THP-1 macrophages. The expression levels of vRNA and IL-1β were quantified by RT-qPCR. Our results showed that the expression of vRNA was increased dose-dependently, while the IL-1β transcripts were not altered (Fig. 7A). The amounts of mature IL-1β protein were analyzed by ELISA. The released IL-1β levels were up-regulated in vRNA-transfected THP-1 macrophages, suggesting that vRNA is sufficient to promote inflammasome activation in macrophages (Fig. 7B). Total RNA extracted from RD cells was used as a control since the EV-A71 virus was propagated in these cells. To determine whether de novo protein synthesis is required for EV-A71-triggered IL-1β production, cycloheximide (CHX) was added to suppress viral protein synthesis in EV-A71-infected cells. Western blot showed that the viral protein expression was drastically decreased in CHX-treated cells (Fig. 7C). The obtained data showed that cycloheximide treatment resulted in an approximate 2/3 reduction in secreted IL-1β, which suggested that the viral protein was not the only factor that triggers IL-1β production (Fig. 7D). Prior research revealed that caspase-8 is essential in TLR3/4-mediated IL-1β production^28^. We found that transfection of virus RNA led to the cleavage of caspase-8 in THP-1 macrophages (Fig. 7E). To further assess whether caspase-8 activation was implicated in RNA-triggered IL-1β production, macrophages transfected with cellular and viral RNA were treated with Z-IETD-FMK. ELISA showed that the release of IL-1β in response to vRNA transfection was drastically suppressed by Z-IETD-FMK treatment (Fig. 7F). Therefore, we revealed a novel mechanism showing that caspase-8 is implicated in EV-A71-mediated IL-1β synthesis in macrophages (Fig. 8).

## Discussion

Inflammasome formation is an innate immune response evoked in host cells in response to stimulation by microbial invasion. Activation of inflammasomes results in the release of the cytokines IL-1β and IL-18, which are implicated in the enhanced production of adhesion molecules and chemokines and the subsequent stimulation of additional inflammatory reactions^34^. Activation of the inflammasome has been previously demonstrated to be associated with the pathogenesis underlying infectious pathogen-related diseases by inducing pyroptosis or neuroinflammation^35,36^. Furthermore, suppression of inflammasome activation can alleviate HIV-mediated neuroinflammation and neuronal injury, which indicates that modulating inflammasome formation may be a therapeutic antiviral strategy^17^.

Viral infection can trigger the production of IL-1β in host cells. The NLRP3 inflammasome is the most common inflammasome activated in cells infected with an RNA virus^37^. For example, flaviviruses, including HCV, West Nile virus (WNV), and Japanese encephalitis virus (JEV), can induce the formation of the NLRP3 inflammasome, leading to increased production of IL-1β^29,38,39^. Furthermore, recent studies have shown that infection by picornaviruses such as rhinovirus, coxsackievirus B3 (CVB3), poliovirus, and EV-A71 results in IL-1β secretion and is also associated with the activation of the NLRP3 inflammasome^16,30,40^. Interestingly, viruses can promote the activation of different inflammasomes. For instance, the AIM2 inflammasome is formed in EV-A71-infected SK-N-SH cells^41^. Our results showed that, in addition to NLRP3, knocking down RIG-I expression also led to a decrease in EV-A71-triggered IL-1β production, suggesting that RIG-I is implicated in EV-A71-induced inflammasome formation. Therefore, our results showed that RIG-I and NLRP3 play roles in EV-A71-induced IL-1β production. Currently, the role of IL-1β in virus-infected pathogenesis is not clear. Previous studies have shown that IL-1β influences West Nile virus infection in dendritic cells^42^. Wang et al. indicated that IL-1β induces tissue Damaged and leaky blood vessels in vivo^43^. IL-1β mediated inflammation, fever and fibrosis in SARS-CoV-2-infected lung has been reported^44^. Therefore, IL-1β is a potential target in viral infection therapy.

RIG-I can interact with ASC and subsequently trigger inflammasome formation in human peripheral blood mononuclear cells in a caspase-1-dependent manner to produce mature IL-1β. Previous research pointed out that RIG-I, the cytosolic RIG-I-like receptor (RLR) sensor, can transmit signals that activate inflammasomes and drive subsequent IL-1β production^45^. Human primary bronchial epithelial cells generate IL-1β through the interaction of RIG-I with ASC and caspase-1 upon infection with influenza A ^14^. A similar result was observed in HCV-infected cells, in which both RIG-I and NLRP3 played roles in inflammasome activation^46^. Only a few viruses, including VSV and IAV, have been reported to trigger IL-1β production through RIG-I inflammasome formation^14,45^. The RIG-I inflammasome is activated in primary lung epithelial cells and peripheral blood mononuclear cells. Furthermore, the activation of the epithelial RIG-I inflammasome is implicated in virus-induced inflammatory responses^47^.

Previous studies have shown that RIG-I mediates inflammasome activation in a caspase-1-dependent manner^13,45^. Interestingly, our results revealed that caspase-8 is also involved in RIG-I agonist-triggered IL-1β production in human macrophages. Z-IETD-FMK, a caspase-8 inhibitor, and Ac-YVAD-cmk, a caspase-1 inhibitor, can serve as potent reagents for suppressing RIG-I engagement-induced IL-1β release^48,49^. Therefore, both caspases play essential roles in mediating the activation of the RIG-I inflammasome. Caspase-8 is a caspase protein that acts as an initiator molecule in the extrinsic apoptosis pathway^50^. Recent research determined that caspase-8 is involved in the induction and maturation of IL-1β in specific cell types, including dendritic cells and macrophages^51,52^. Caspase-8 is involved in NLRP3 inflammasome activation in these cells^53,54^. Caspase-8 induces alternative inflammasome activation in human monocytes and human tissue macrophages^55,56^. Through the interaction with FADD, caspase-8 can activate NLRP3 activation, which is a species-specific response^55^. Previous research revealed that caspase-8 is essential in mediating TLR3- and TLR4-mediated IL-1β production^28^. Although recruitment of caspase-8 is implicated in Salmonella-induced inflammasome activation, there is no report demonstrating that viral pathogens can induce IL-1β release by caspase-8^54^. Our results revealed that caspase-8 is related to activating RIG-I and NLRP3 inflammasomes in human macrophages, showing that caspase-8 is essential for EV-A71-induced IL-1β release.

The mechanisms underlying virus-induced IL-1β production have been intensively studied. Accumulated evidence indicates that viral proteins are essential in activating inflammasome formation in host cells. For example, viral protein 2B in EMCV, PV, and EV-A71, as well as envelope (E) protein from SARS-CoV, are known to activate the NLRP3 inflammasome by inducing a flux of calcium ions^16,57^. Other viruses, such as SARS-CoV, can cause the formation of the NLRP3 inflammasome via the E protein by changing the distribution of Na + /K + ^58^. In addition to virus-encoded proteins, virus-derived nucleic acid derivatives can also trigger inflammasome formation. IAV and HCV genomic RNA could result in NLRP3 inflammasome activation to produce mature IL-1β^59,60^. Nevertheless, whether RNA species derived from picornaviruses can trigger IL-1β maturation is controversial. Previous studies have shown that EV-A71 genomic RNA fails to induce IL-1β production, while foot-and-mouth disease virus RNA can trigger NLRP3 inflammasome activation^61,62^. The discrepancy in experimental results might come from the differences in cell models and methods. In this study, we utilized human macrophages as our cell model and thus observed species-specific caspase-8-mediated inflammasome activation processes.

Our results showed that RIG-I, a cytosolic RNA sensor, is implicated in EV-A71-induced IL-1β maturation, indicating that PRRs can mediate the activation of inflammasomes in human macrophages. In addition to RIG-I, our results showed that TLR3 and TLR8 are involved in EV-A71-triggered IL-1β release. TLR stimulation can result in ATP secretion and thus activate the P2X7 receptor to induce the secretion of mature IL-1β in primary human monocytes^63,64^. TLR3, a type I transmembrane protein localized in the endosome, serves as a sensor for recognizing dsRNA. Recent research shows that TLR3 engagement can promote NLRP3 inflammasome activation via TRIF/RIPK1/FADD-dependent pathways^33^. In addition, TLR3 contributes to IAV RNA-induced IL-1β production in human bronchial epithelial cells^14^. TLR8 is a ssRNA-recognizing RNA sensor that resides in endosomes and is responsible for recognizing various RNA viruses, such as IAV and HIV^32,65,66^. Recent studies have revealed that TLR8 stimulation involves the activation of the NLRP3 inflammasome in human monocytes^64,67^. Furthermore, Plasmodium RNA stimulates the release of IL-1β and IL-18 from human PBMCs via TLR8^68^. Our results showed that the knockdown of TLR3 and TLR8 resulted in the downregulation of EV-A71-induced IL-1β production. The IL-1β induced by engagement with TLR3 and TLR8 depends on NLRP3, caspase-1, and caspase-8. Previous studies showed that TLR3 and TLR8 ligands could stimulate the release of IL-1β via the activation of the NLRP3 inflammasome^33,64,67,69^. A more recent study implicated caspase-8 in SARS-CoV-2 ssRNA-induced NLRP3 inflammasome activation, indicating that caspase-8 is involved in TLR8-mediated IL-1β production in human macrophages^70^. Thus, TLR ligation can promote NLRP3 inflammasome activation via a caspase-8-dependent pathway.

Growing evidence indicates that RNA species can activate IL-1β production by promoting the formation of multiple inflammasomes. Different RNA species, such as replicative RNA intermediates and digested RNA products, can trigger inflammasome formation and produce IL-1β. For instance, Rajan et al. demonstrated the activation of the NLRP3 inflammasome by poly(I:C) transfection^71^. Furthermore, poly(I:C), feline herpesvirus (FHV) dsRNA, and HCV genomic RNA can induce myeloid cells to produce IL-18 and IL-1β by activating the NLRP3 inflammasome^19,60^. However, contradictory results have been reported by Ichinohe et al., whose data indicated that transfection of viral genomic RNA or poly (I:C) into murine bone marrow-derived macrophages did not induce IL-1β production^65^. RNA species derived from CVB3 and FHV cannot stimulate the secretion of IL-1β in mouse macrophages^19,20^. The mechanism underlying RNA-triggered IL-1β production in macrophages is species specific, which can explain the discrepancy.

We demonstrated that EV-A71-induced IL-1β maturation in human macrophages depends on NLRP3, RIG-I, caspase-1, and caspase-8. The caspase-8 inhibitor can decrease the production of IL-1β, which results from the activation of NLRP3 and RIG-I inflammasomes. Furthermore, the expression of TLR3 and TLR8 can influence IL-1β release in an NLRP3-dependent manner, suggesting that RNA sensors are implicated in EV-A71-triggered IL-1β production. Transfection of genomic RNA can result in IL-1β maturation in THP-1 macrophages via the caspase-8-dependent pathway. In conclusion, caspase-8 is essential in mediating EV-A71-triggered IL-1β production, and PAMPs, such as RIG-I, TLR3, and TLR8, also contribute to inflammasome activation.

## Material and methods

### Cells and virus

THP-1 cells were cultured in RPMI-1640 medium (Thermo-Fisher Scientific, MA, USA) containing 10% fetal bovine serum (FBS) (GE, Boston, USA). NLRP3-KD THP-1 cells (a kind gift from Dr. Yang, Chang Gung University, Immunology Center) are THP-1 cells that are infected with lentiviral vectors that carried NLRP3 shRNA. Human rhabdomyosarcoma (RD) cells were obtained from Dr. Shih Shin-Ru and grown in Dulbecco modified Eagle medium (DMEM) containing 10% FBS, 1% non-essential amino acids, 1% L-glutamine, and 1% penicillin/streptomycin (all from Thermo-Fisher Scientific, MA, USA). All cells were incubated in a 37 °C incubator that was equilibrated with 5% CO2. The EV-A71 strain 2231 (TW/2231/98) was isolated from the Clinical Virology Laboratory of Chang Gung Memorial Hospital (Linkou, Taiwan), was amplified using RD cells, and the titer was quantified by plaque forming assays.

### Isolation of PBMCs and differentiation of PBMCs toward macrophages

Human peripheral blood samples were collected after approval by IRB (Chang Gung Medical Foundation institutional review board, IRB2019050059). All the research process was performed in accordance with IRB guidelines and regulations. The informed consent was obtained from all subjects and/or their legal guardians. Mononuclear cells were harvested by Ficoll-Paque method. Briefly, peripheral blood was mixed with PBS (1:1). The diluted cell suspension was then layered on Ficoll-Paque (GE Healthcare Life Sciences, MA, USA) (volume 2:1) in a 50 mL canonical tube. After centrifugation at 400 × g for 20 min, the top layer was aspirated. The mononuclear cell layer in the interface was then transferred to a new tube. To differentiate the harvested PBMCs toward macrophages, RPMI medium supplemented with 20 ng/mL M-CSF (Peprotech, NJ, USA) and 1% human serum was added and cultured for 5 days.

### Reagents

Phorbol 12-myristate 13-acetate (PMA) was prepared at 200 µM in DMSO (Dimethyl sulfoxide). 200 nM PMA was used to stimulate THP-1 cell differentiation. Ac-YVAD-cmk (Sigma-Aldrich, MO, USA) was prepared at 10 mM in DMSO. 1 µM Ac-YVAD-cmk was used to inhibit caspase-1 activation. Z-IETD-FMK (R&D systems, MN, USA) was prepared at 20 mM in DMSO. 20 µM Z-IETD-FMK was used to treat PMA primed THP-1 after virus infection. Motolimod was dissolved at 1 mM in DMSO and simulated the cells at concentration of 10 µM.

### Virus infection

Cells were seeded in culture plates and cultivated at different time points. The seeded cells were rinsed with PBS twice and serum-free medium was added with the virus at a specified multiplicity of infection (M.O.I.). Adsorption was performed at 37 °C for 1 h, and the medium was then decanted. The cells were washed with PBS twice and 2% FBS containing medium was added for further incubation.

### RNA extraction and RT-qPCR

Trizol reagent (Life Technologies, Gaithersburg, MD) was applied to extract total RNA from cell samples. The cells were lysis by Trizol reagent and then mixed with chloroform. After 5 min, the homogenate was centrifugated at 12,000 × g for 15 min at 4 °C. The aqueous phase was transferred to a new tube and added with an equal volume of isopropanol and then incubated for 10 min. The mixture was centrifuged at 12,000 × g for 8 min at 4 °C. After the centrifugation, the supernatant was removed. 500 µL 70% ethanol was used to wash the RNA pellet then centrifugated at 12,000 × g for 5 min at 4 °C. Discard the supernatant and then air dry the RNA pellet. The RNA pellet was dissolved in sterile water and quantified the concertation of RNA by NonoDrop technology (Thermo-Fisher Scientific, MA, USA). 1 µg of total RNA was used to synthesize cDNA. RevertAid First Strand cDNA Synthesis Kit (Thermo-Fisher Scientific, MA, USA) was applied according to the manufacturer's instructions. To detect the expression of target genes, specific primers for IL-1β and EV-A71 5’ untranslated region (5’UTR) were used (Table 1). qPCR assays were carried out on 384-well plates and analyzed by a Roche Lightcycler 480 instrument (Roche, Basel, SW). Triplicate for each sample in qPCR analysis and 18s rRNA was used as a reference gene. The relative expression level of each gene was analyzed by 2^−ΔΔCT^ method.

**Table 1:** qPCR primer used in this study.

Gene		Sequence
Human 18s rRNA	Forward	5’-GTA ACC CGT TGA ACC CCA TT -3’
	Reverse	5’-CCA TCC AAT CGG TAG TAG CG -3’
EV-A71 5’UTR	Forward	5’-CCC TGA ATG CGG CTA ATC C -3’
	Reverse	5’-ATT GTC ACC ATA AGC AGC CA -3’
Human IL-1β	Forward	5’-ACA GAT GAA GTG CTC CTT CCA -3’
	Reverse	5’-GTC GGA GAT TCG TAG CTG GAT -3’

### Protein isolation and western blot

The cultured cells were harvested at indicated time point. After washed with PBS twice, the cell lysates were collected using protein lysis buffer (1% NP-40, 50 mM Tris, and 150 mM NaCl) containing protease inhibitors cocktail (Bioshop, Ontario, Canada). After incubated on ice for 30 min, centrifugation was performed at 13,000 rpm for 10 min at 4 °C. The Supernatants were harvested, and protein concentrations were measured by Protein Assay Dye Reagent (Bio-Rad Laboratories, CA, USA). Proteins (30 µg) were separated by 10% or 12% SDS–polyacrylamide gel electrophoresis and then transferred onto a polyvinylidene fluoride membrane (PVDF) (GE, MA, USA). The membrane was then blocked with 5% skim milk in Tris-buffered saline Tween-20 buffer (TBST) (20 mmol/L Tris–HCl, pH 7.4, 150 mmol/L NaCl, and 0.1% Tween 20) at room temperature for one hour. After washed by TBST twice, the membrane was then incubated with anti-EV-A71 3D Ab (1:10,000, was gifted by Professor Shih Shin-Ru), anti-EV-A71 VP0 (1:1000, Millipore, MA, USA), anti-IL-1β Ab (1:1000, Cell Signaling Technology, MA, USA), anti-NLRP3 Ab (1:1000, Cell Signaling Technology, MA, USA), anti-ASC Ab (1:2000) (1:1000, Santa Cruz Biotechnology, TX, USA), anti-caspase-1 Ab (1:1000, Santa Cruz Biotechnology, TX, USA), anti-caspase-8 Ab (1:1000, Cell Signaling Technology, MA, USA), Anti-RIG-I Ab (1:1000, Enzo Life Sciences, NY, USA), anti-TLR3 Ab (1:1000, Abcam, CAMB, UK) and anti-TLR8 Ab (1:1000) (Invitrogen, CA, USA)., The unbound antibodies were then removed by washing with TBST three times. Horseradish peroxidase-conjugated secondary antibodies (1:5000, Jackson ImmunoResearch Laboratories, Pennsylvania, USA) were incubated for 1 h. Finally, the target proteins were visualized with a Western Lightning Chemiluminescence reagent (PerkinElmer, MA, USA) and detected by ChemiDoc imaging system (Bio-Rad Laboratories, CA, USA).

### Co-immunoprecipitation

Total protein was harvested by IP lysis buffer (1% NP-40, 50 mM Tris, and 150 mM NaCl). 1 mg protein samples were incubated with 2 μL anti-NLRP3 or anti-ASC and then rotated at 4 °C overnight. After incubation, protein A and protein G agarose beads (all purchased by Blossom Biosciences, CA, USA) were added into the prepared samples and then rotated for 4 h at 4 °C. After the hybridization, Immunoprecipitates were washed by wash buffer (20% NP40, 1 M Tris–HCl pH 7.5, 5 M NaCl) thrice. Protein samples were eluted by protein loading buffer (100 mM Tris–HCl, 10% glycerol, 0.1 M DTT, 2% SDS, and 0.1% bromophenol blue) and analyzed by immunoblot.

### Extraction of viral RNA and transfection

Viral RNA was isolated from virions or RD cells infected with EV-A71 by QIAamp Viral RNA Kits (Qiagen, Hilden, Germany). The RNA was quantified by NanoDrop technology (Thermo Scientific). Cells were transfected with viral RNA, RD RNA, 5’ppp-dsRNA or poly(A:U) using Lipofectamine 2000 (LF2K) reagent (Invitrogen, CA, USA).

### siRNA transfection

The siRNAs targeting NLRP3, caspase-1, RIG-I, and TLR3, as well as scramble siRNA, were used in this study (all from Sigma-Aldrich, MO, USA). The siRNA was prepared in 20 μM with RNase-free distilled water. 100 nM siRNA was used to transfect into the cells with Lipofectamine RNAiMAX (Thermo-Fisher Scientific, MA, USA) in opti-MEM medium (Thermo-Fisher Scientific, MA, USA) for 48 h. After transfection, the cells were infected with EV-A71 and harvested the sample at various hours post-infection.

### Lentivirus preparation and gene knockdown

The Lentivirus-based shRNA constructs used to target human TLR8 were obtained from the National RNAi Core Facility (Taiwan) and negative control of shRNA construct (shNC) was obtained from Dr. Shih Shin-Ru (Chang Gung University, Taiwan). To prepare for lentivirus production, the HEK-293 T cells were transfected with pMD. G, pCMVΔ8.91 and pLKO-shRNA, with TransIT-LT1 transfection reagent (Mirus Bio, WI, USA) for 48 h. The supernatant was harvested after centrifugation at 4000 rpm for 10 min at 4 °C. The stable phenotype of shRNA knockdown THP-1 cells was selected by 1 µg/mL puromycin after lentivirus infection for 48 h.

### Plaque assay

The total virus was harvested at different time points after virus infection. RD cells were seeded on 6-well plate at a density of 5 × 10^5^ cells per well. After incubation, the collected virus was serially diluted by 0% FBS DMEM medium. RD cells were washed with PBS twice and then infected by diluted virus medium. After absorption, the medium was removed and replaced by 2% FBS DMEM medium containing 0.3% agarose. After 96 h of incubation, the medium was removed and the cells were stained by crystal violet.

### ELISA

The culture medium was collected at different time points. Centrifugation was performed at 5,000 rpm for 10 min at 4 °C then the supernatants were harvested. IL-1β ELISA kit was obtained from Invitrogen (Thermo-Fisher Scientific, MA, USA). The anti-IL-1β antibody was coating on the 96 well plate overnight at 4 °C. After incubation, 96-well plate was washed by washing buffer once. Blocking the 96 well plate by assay buffer for 1 h. Aspirate wells and then 100 μL standards and samples were added into each well with detection antibody. The 96-well plate was incubated at 700 rpm for 2 h at room temperature. Aspirate wells and then wash by wash buffer five times. The working streptavidin-HRP was added into each well and then incubated at 700 rpm for 30 min at room temperature. The plate was washed five times. TMB (3, 3', 5, 5'-tetramethylbenzidine) solution was added into each well. Stop the reaction using 2 M H_2_SO_4_ solution. Detect the absorbance at 450 nm and 650 nm by microplate reader (BioTek, VT, USA).

### Statistical analysis

All experiments were repeated at least 3 times. Results were shown as the means ± SD. The data were analyzed by Student’s unpaired T-test. The value of *p* < 0.05 was indicated statistical significance.

## Supplementary Information

Supplementary Information 1.

Supplementary Information 2.

Supplementary Information 3.

Supplementary Information 4.

Supplementary Information 5.

## Data Availability

The datasets used and analyzed during the current study are available from the corresponding author on reasonable request.
