# Supplementary material for: EV-A71 induced IL-1β production in THP-1 macrophages is dependent on NLRP3, RIG-I, and TLR3
Source: Sci Rep. 2022 Dec 11;12:21425. doi: 10.1038/s41598-022-25458-x (PMC9741760; doi:10.1038/s41598-022-25458-x)
Supplement: Supplementary file 4 — Supplementary Information 4. [file 41598_2022_25458_MOESM4_ESM.docx]

**
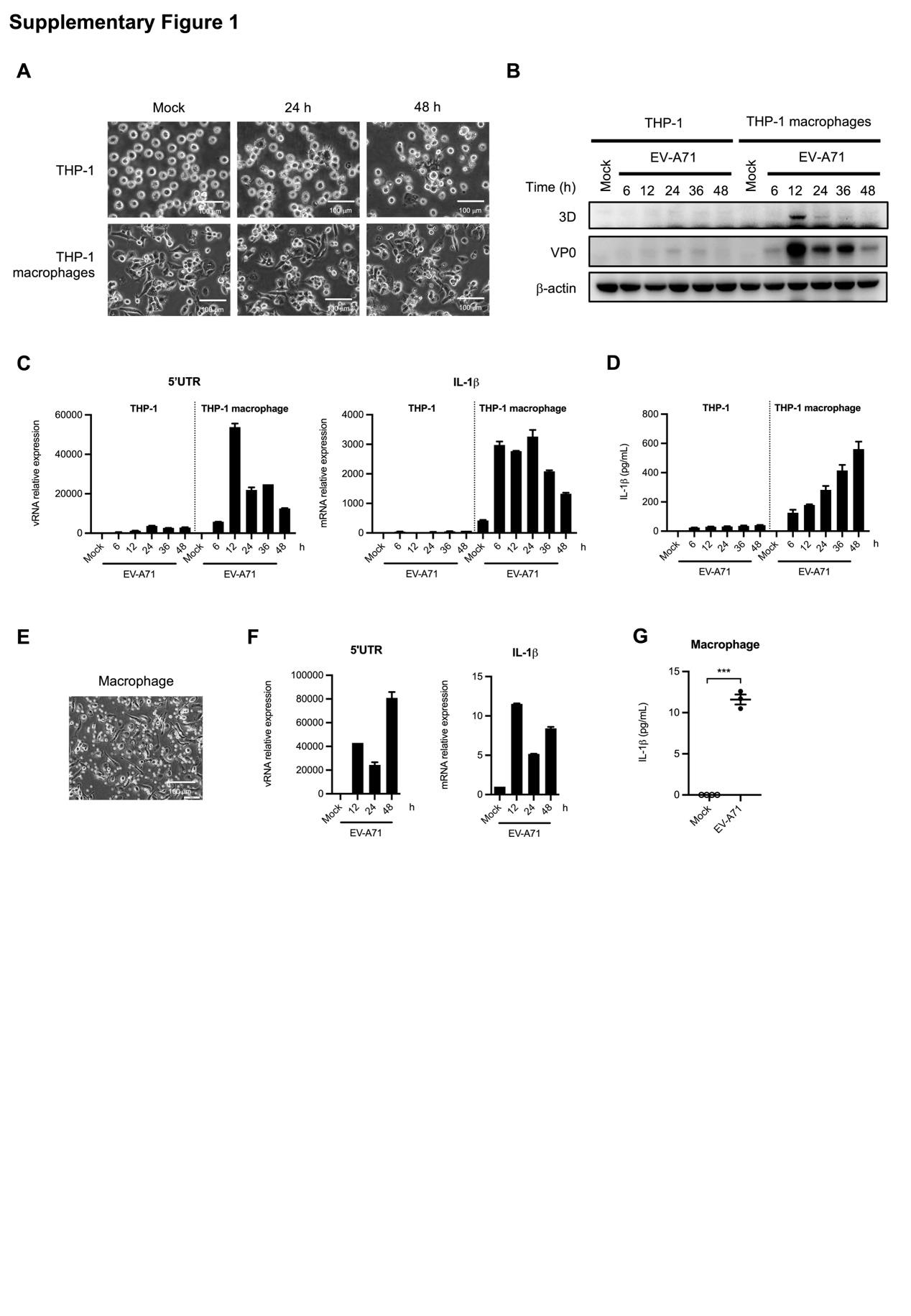
**

**Supplementary Figure 1. EV-A71 infection induces IL-1β production in macrophages.** THP-1 and THP-1 macrophages were infected with EV-A71 at an M.O.I. of 2. (A) The morphologies of infected cells were observed using inverted microscopy, and images were taken at 24 and 48 hours post-infection. (magnification = 200X). (B) Total protein was extracted from mock- and EV-A71-infected cells and then subjected to Western blot to examine the expression of viral protein 3D, VP0. The β-actin expressed was used as an internal control. (C) Total RNA was isolated, and then the EV-A71 viral RNA 5’UTR and IL-1β mRNA were detected by RT-qPCR. (D) The secreted amounts of IL-1β in the supernatants were detected by ELISA. Human PBMCs were collected from healthy volunteers and obtained in the presence of 10 ng/mL M-CSF medium for 7 days. (E) The morphologies of differentiated cells were observed using inverted microscopy. (F) Human PBMCs-derived macrophages were infected with EV-A71 at the M.O.I. of 10. Total RNA was collected at 12, 24, and 48 h post-infection. The expression levels of EV-A71 viral RNA 5’UTR and IL-1β mRNA were examined by RT-qPCR. (G) The expression level of IL-1β in supernatant for 24 hours post-infection was measured by ELISA. (***, p < 0.001, Student’s unpaired T-test)


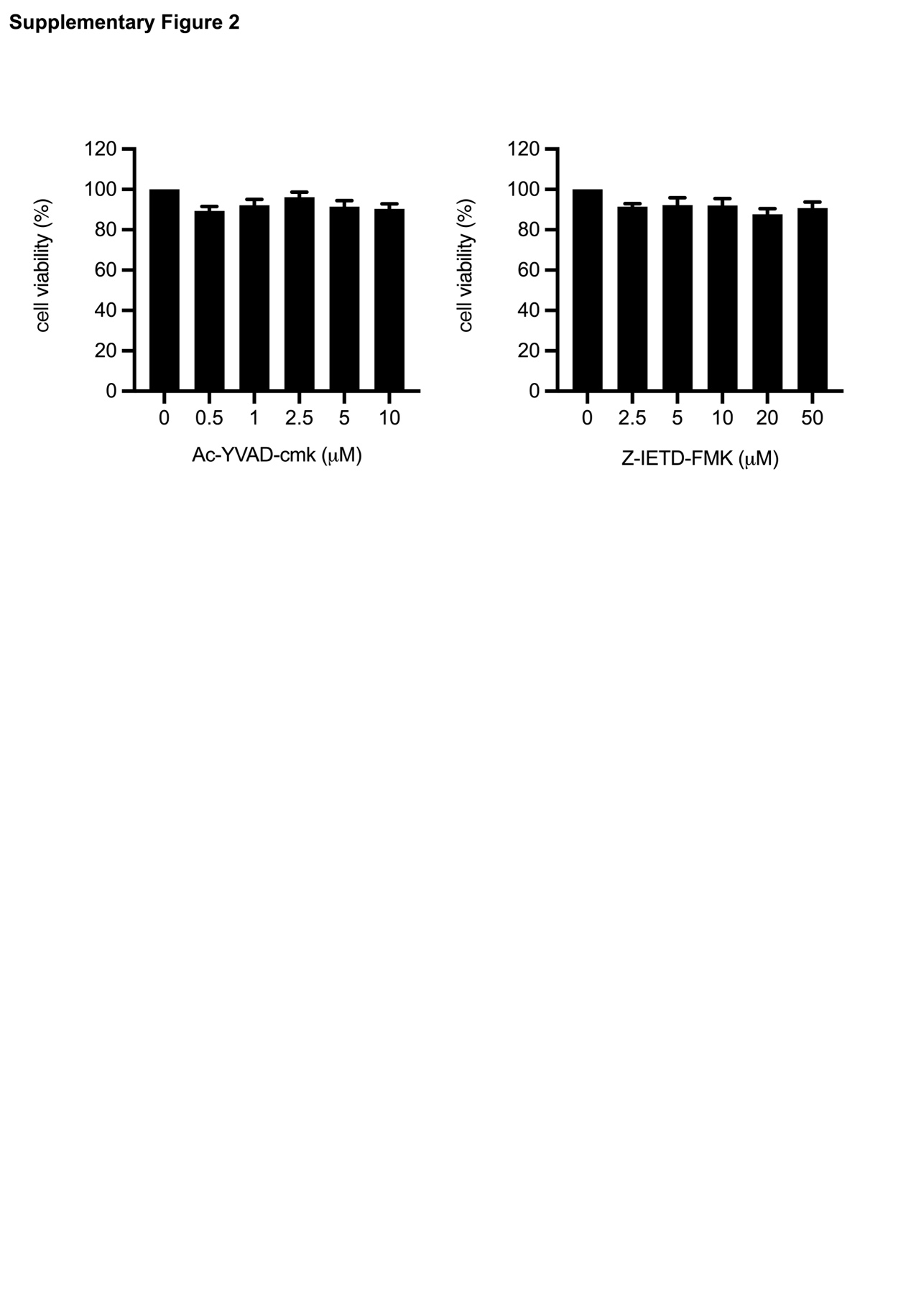


**Supplementary Figure 2. Ac-YVAD-cmk and Z-IETD-FMK treatment exhibit low cytotoxicity in THP-1 macrophages.** THP-1 cells were treated with 200 nM PMA for 48 hr to differentiate into macrophages. After differentiation, the cells were incubated with Ac-YVAD-cmk or Z-IETD-FMK for 72 hr. The cell viability was measured by MTT assay.


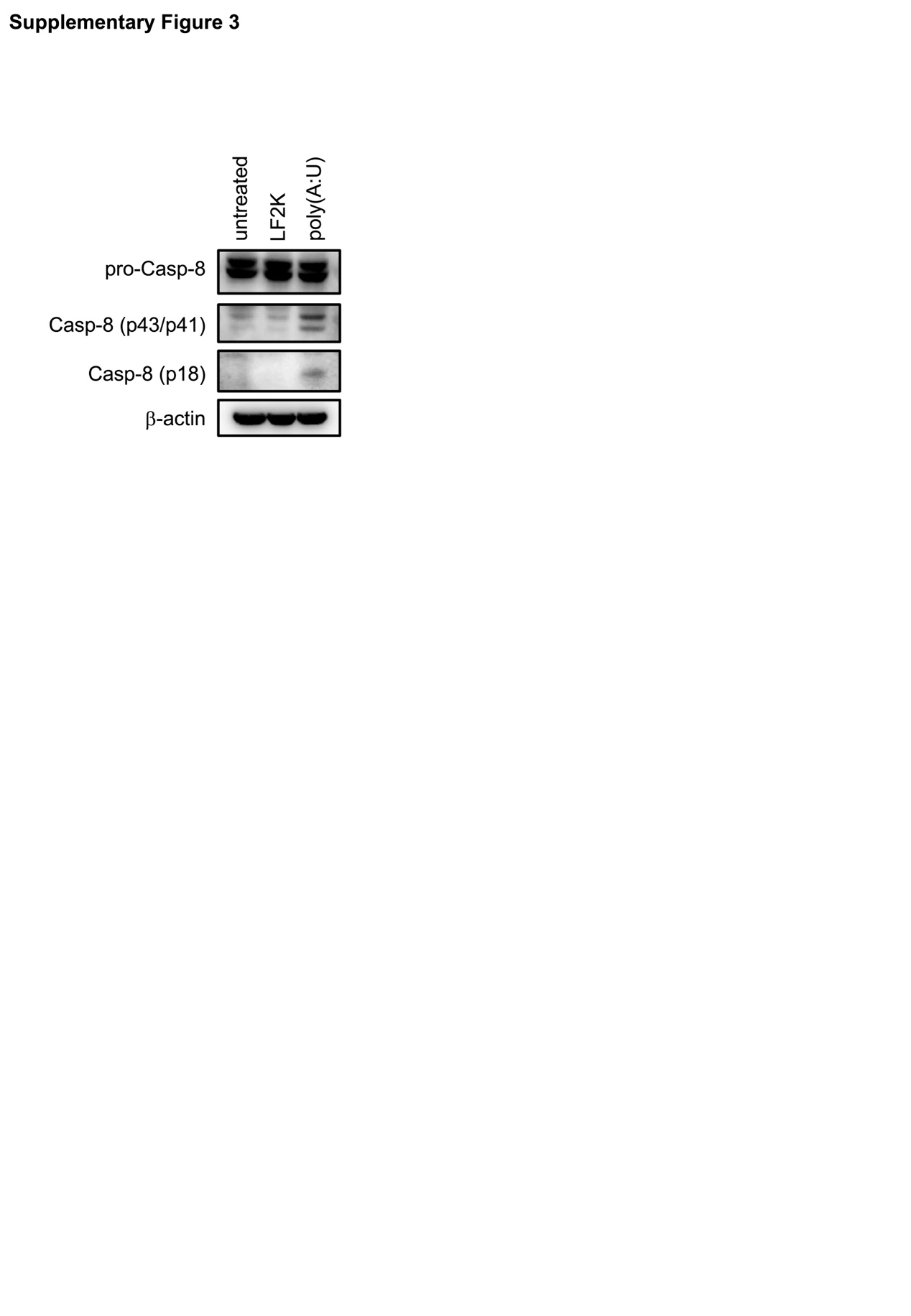


**Supplementary Figure 3. TLR3 agonist induces caspase-8 activation in THP-1 macrophages.** (A) THP-1 macrophages were transfected with 1 μg poly(A:U) for 12 hr. The expression levels of cleaved caspase-8 were analyzed by immunoblot. (B) THP-1 macrophages were transfected with siRIG-I for 48 hr. The transfected cells were treated with poly(A:U). The expression of RIG-I, pro-caspase-8, cleaved caspase-8 (p43/p41), caspase-8 (p18), and β actin was determined by Western blot.
