# Supplementary material for: EV-A71 induced IL-1β production in THP-1 macrophages is dependent on NLRP3, RIG-I, and TLR3
Source: Sci Rep. 2022 Dec 11;12:21425. doi: 10.1038/s41598-022-25458-x (PMC9741760; doi:10.1038/s41598-022-25458-x)

# EV-A71 induced IL-1β production in THP-1 macrophages is dependent on NLRP3, RIG-I, and TLR3

Hsing-I Huang[1,2,3,4,*,ǂ], Chi-Chong Chio[1,2,3,ǂ], Jhao-Yin Lin[1,2], Chia-Jung Chou[2], Chia-Chen Lin[2], Shih-Hsiang Chen[5,6] and Liang-Sheng Yu[2]

## Figure 1A

| | |
|---|---|
| 1 | PMA_THP-1_Mock |
| 2 | PMA_THP-1_EV-A71_12 hr |
| 3 | PMA_THP-1_EV-A71_24 hr |
| 4 | PMA_THP-1_EV-A71_48 hr |

1 2 3 4

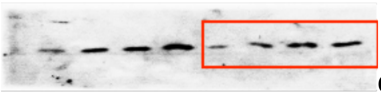

Casp-1 (p20)

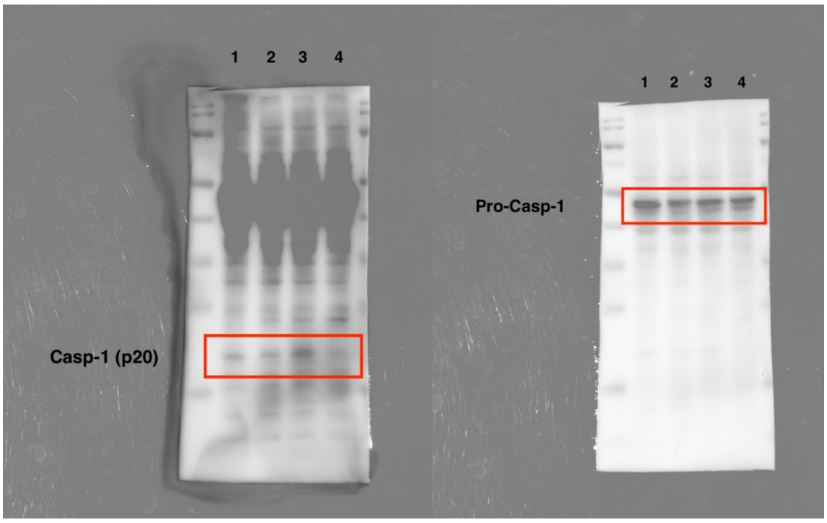


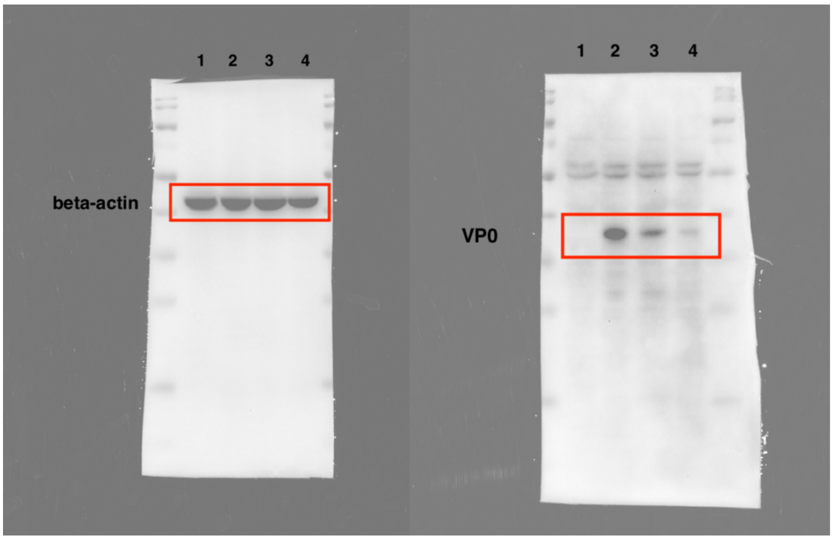

## Figure 1C

| | |
|---|---|
| 1 | PMA_THP-1_Mock |
| 2 | PMA_THP-1_EV-A71_6 hr |
| 3 | PMA_THP-1_EV-A71_12 hr |
| 4 | PMA_THP-1_EV-A71_24 hr |

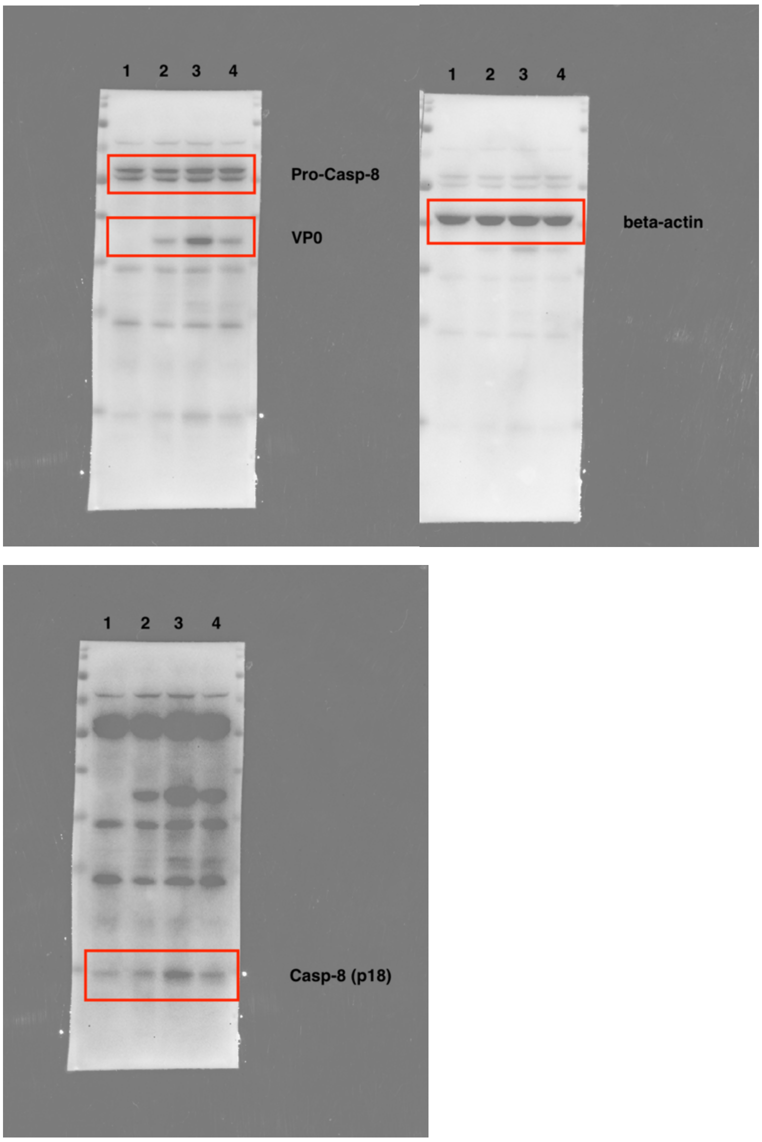

## Figure 2A

| | |
|---|---|
| 1 | PMA_THP-1_Scrambled siRNA_Mock |
| 2 | PMA_THP-1_Scrambled siRNA_EV-A71_12 hr |
| 3 | PMA_THP-1_Scrambled siRNA_EV-A71_24 hr |
| 4 | PMA_THP-1_siNLRP3_Mock |
| 5 | PMA_THP-1_siNLRP3_EV-A71_12 hr |
| 6 | PMA_THP-1_siNLRP3_EV-A71_24 hr |

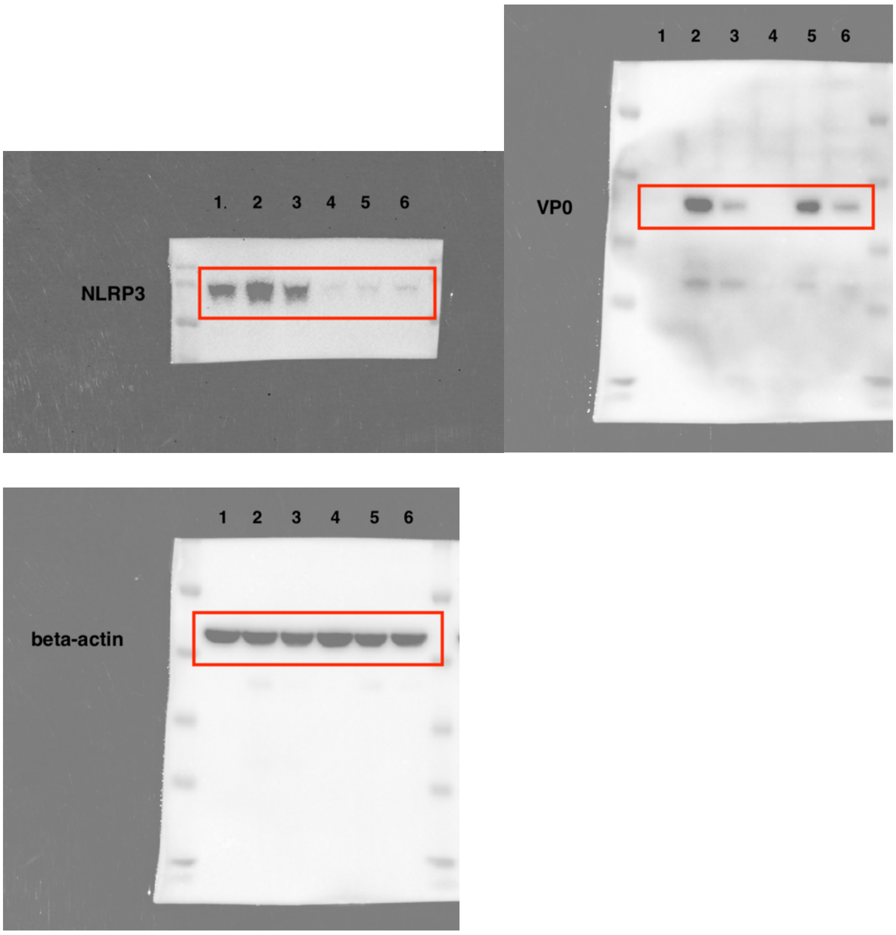

**Figure 2C**

| | |
|---|---|
| 1 | PMA_THP-1_Scrambled siRNA_Mock |
| 2 | PMA_THP-1_Scrambled siRNA_EV-A71_12 hr |
| 3 | PMA_THP-1_Scrambled siRNA_EV-A71_24 hr |
| 4 | PMA_THP-1_siRIG-I_Mock |
| 5 | PMA_THP-1_siRIG-I_EV-A71_12 hr |
| 6 | PMA_THP-1_siRIG-I_EV-A71_24 hr |

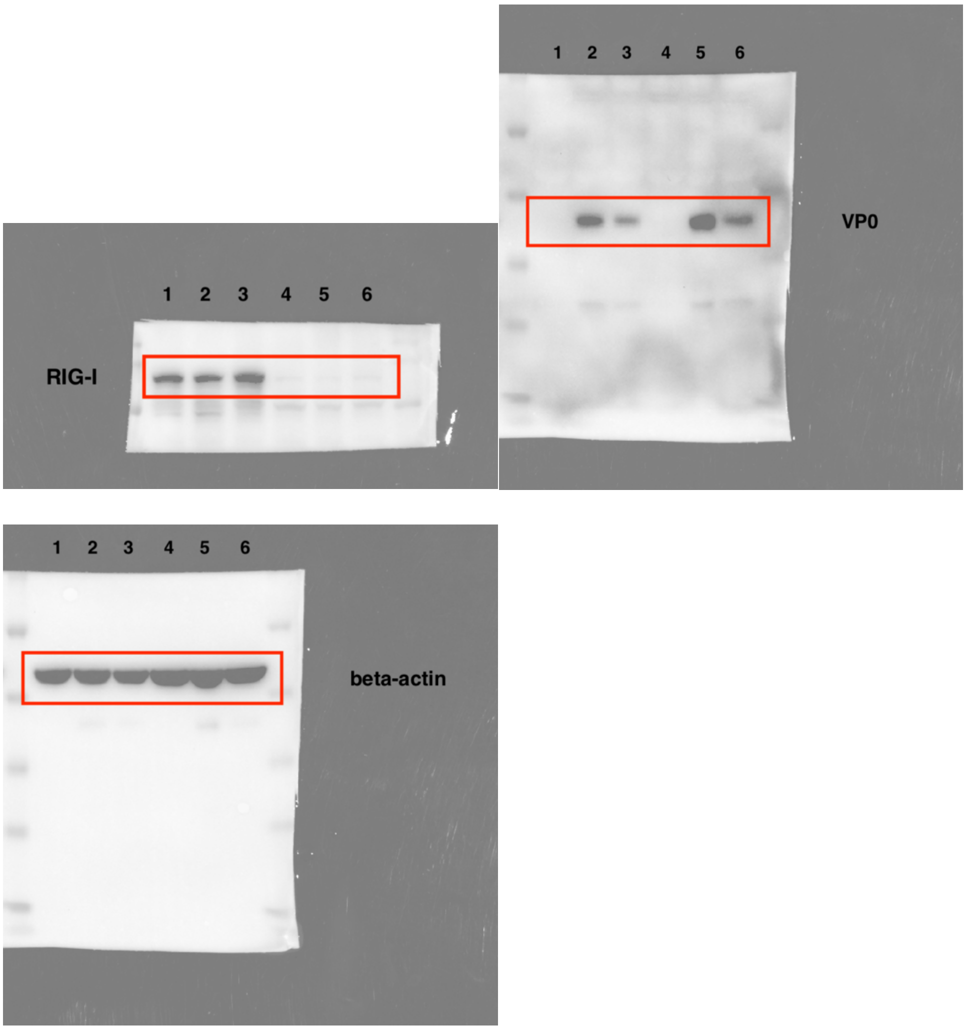

## Figure 2E

| | |
|---|---|
| 1 | PMA_THP-1_Scrambled siRNA_Mock |
| 2 | PMA_THP-1_Scrambled siRNA_EV-A71_12 hr |
| 3 | PMA_THP-1_Scrambled siRNA_EV-A71_24 hr |
| 4 | PMA_THP-1_siASC_Mock |
| 5 | PMA_THP-1_siASC_EV-A71_12 hr |
| 6 | PMA_THP-1_siASC_EV-A71_24 hr |

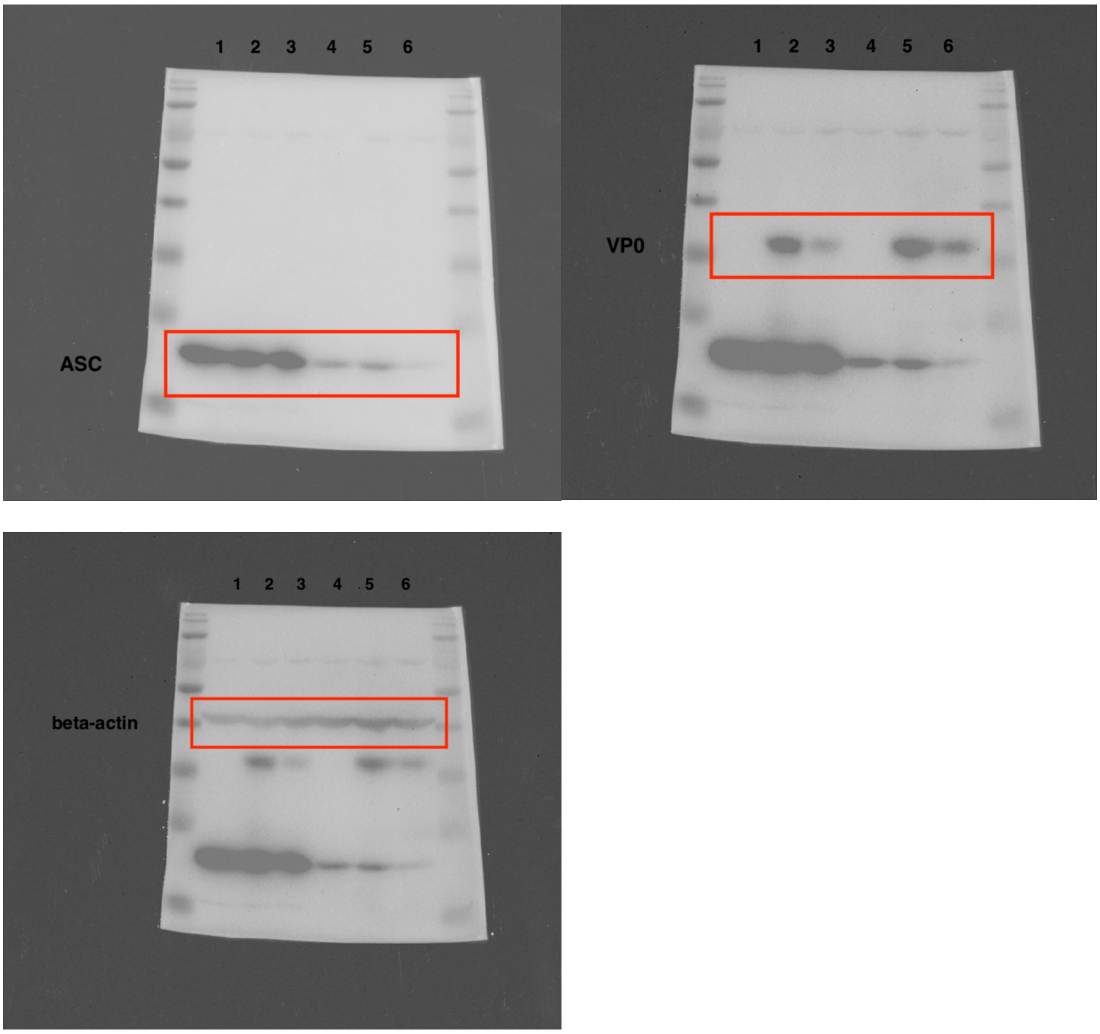

# Figure 3A

| | |
|---|---|
| 1 | PMA_WT THP-1_Mock |
| 2 | PMA_NLRP3 KD THP-1_Scrambled siRNA_Mock_12 hr |
| 3 | PMA_ NLRP3 KD THP-1_Scrambled siRNA_EV-A71_12 hr |
| 4 | PMA_ NLRP3 KD THP-1_siRIG-I_Mock_12 hr |
| 5 | PMA_ NLRP3 KD THP-1_siRIG-I_EV-A71_12 hr |

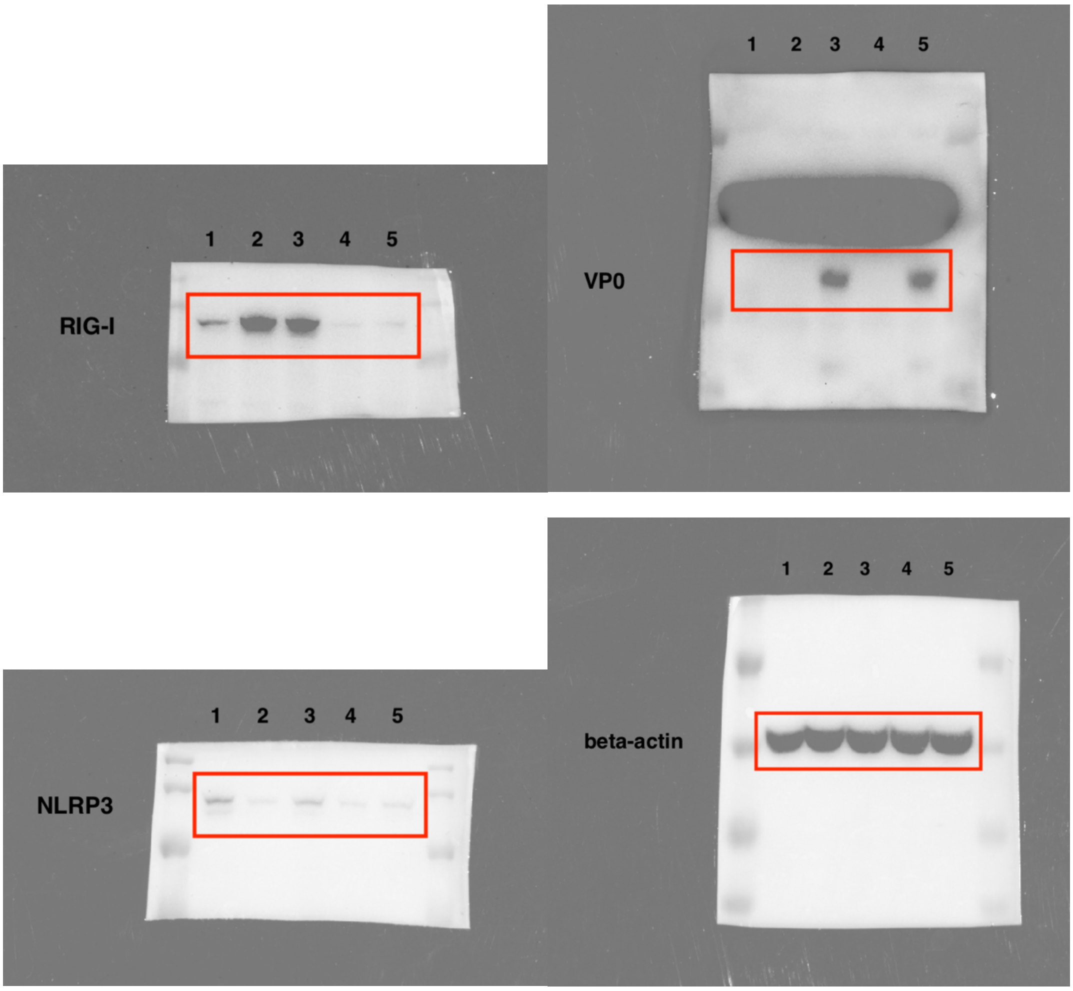

## Figure 3C

| | |
|---|---|
| 1 | PMA_THP-1_Scrambled siRNA_Mock |
| 2 | PMA_THP-1_siRIG-I_ Mock |
| 3 | PMA_THP-1_siNLRP3_ Mock |
| 4 | PMA_THP-1_siRIG-I + siNLRP3_Mock |
| 5 | PMA_THP-1_Scrambled siRNA_EV-A71 |
| 6 | PMA_THP-1_siRIG-I_ EV-A71 |
| 7 | PMA_THP-1_siNLRP3_ EV-A71 |
| 8 | PMA_THP-1_siRIG-I + siNLRP3_ EV-A71 |

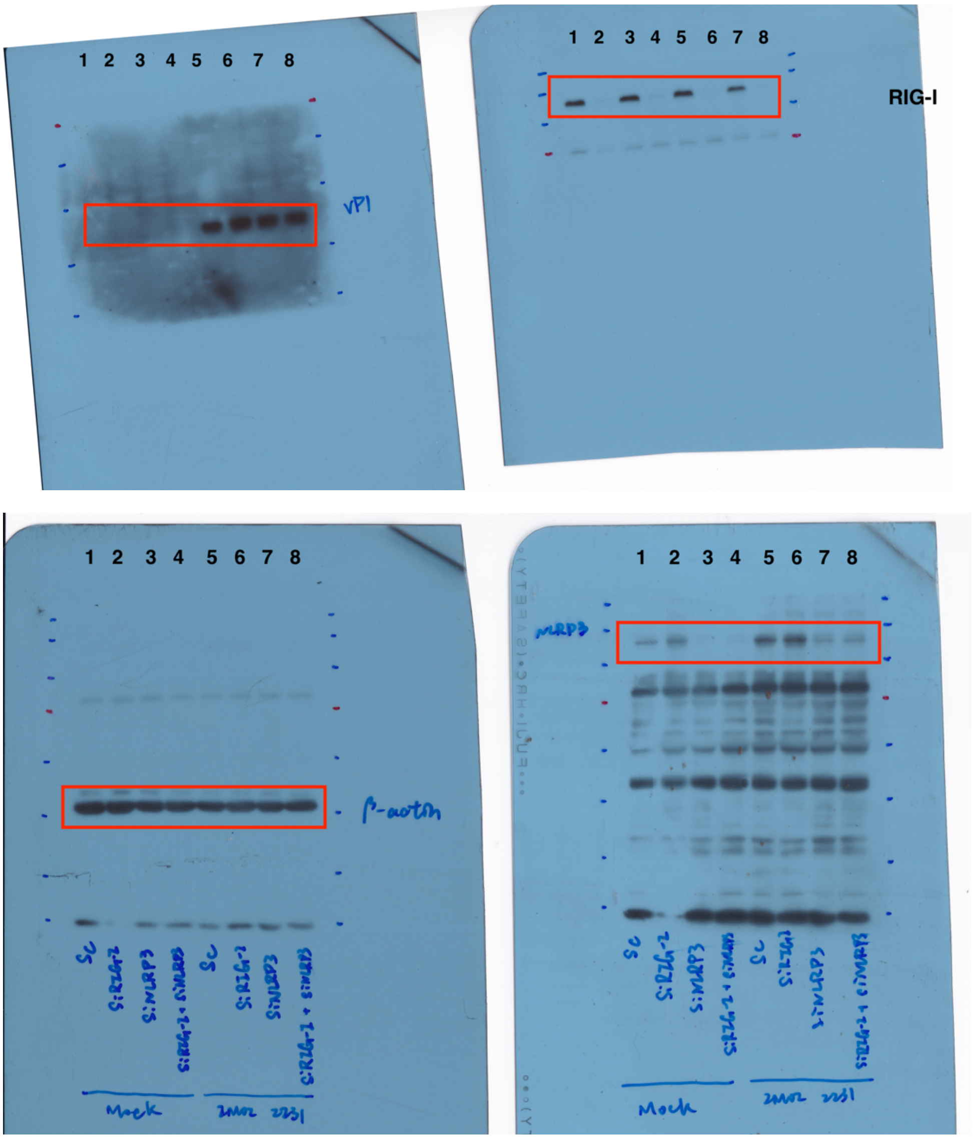

## Figure 3D

| | |
|---|---|
| 1 | PMA_THP-1_Mock |
| 2 | PMA_THP-1_EV-A71 |
| 3 | IP: PMA_THP-1_ IgG control |
| 4 | IP: PMA_THP-1_Mock |
| 5 | IP: PMA_THP-1_EV-A71 |

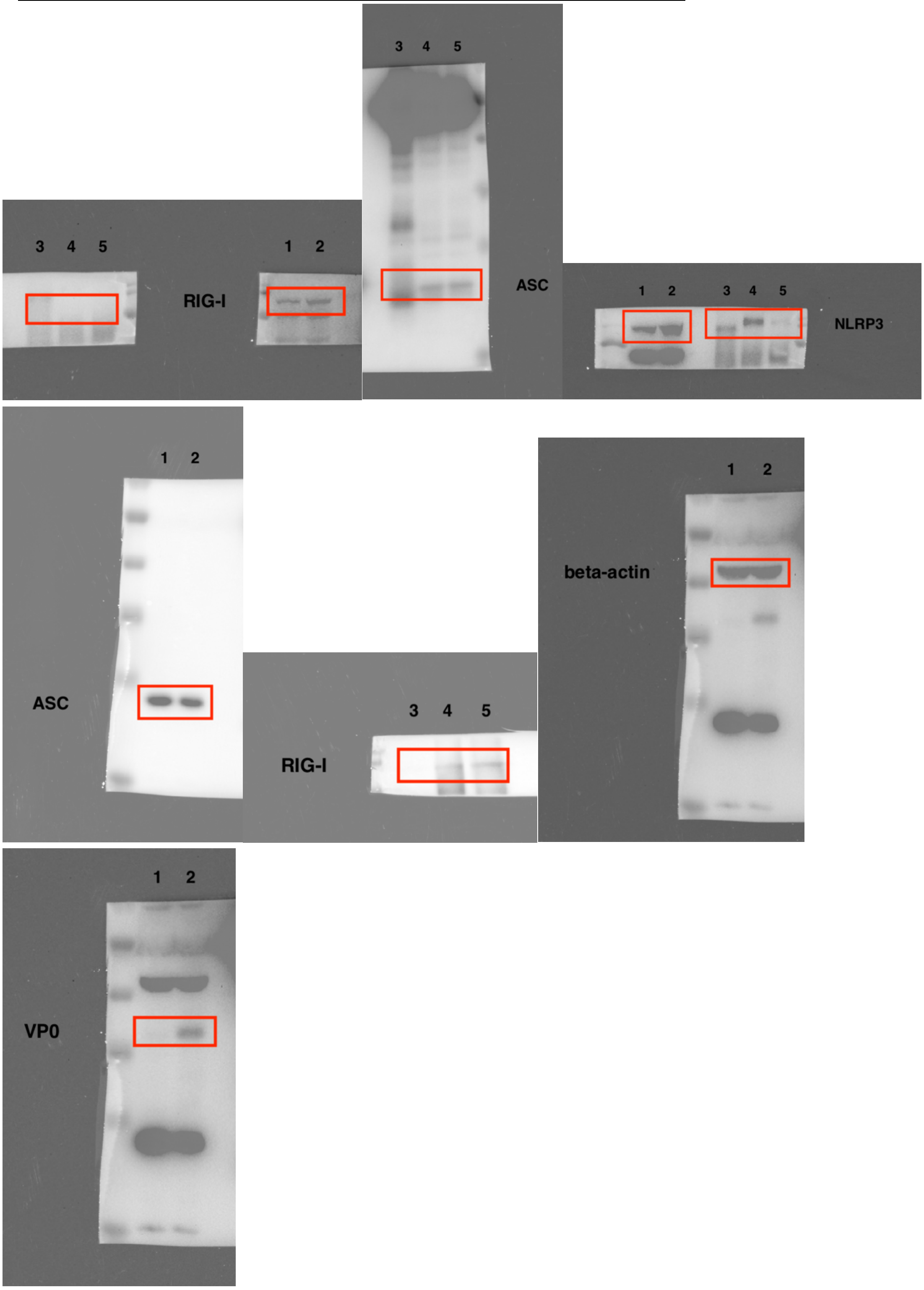

## Figure 4A

| | |
|---|---|
| 1 | PMA_THP-1_scrambled siRNA |
| 2 | PMA_THP-1_siRIG-I |

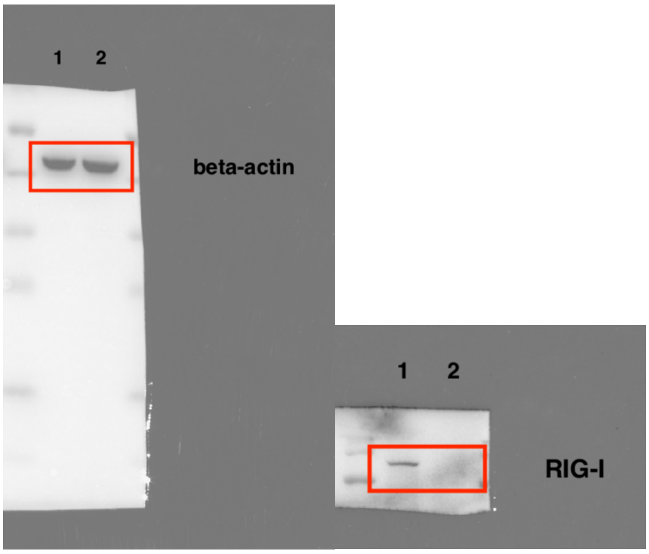


## Figure 4B

| | |
|---|---|
| 1 | PMA_WT THP-1 |
| 2 | PMA_NLRP3 KD THP-1 |

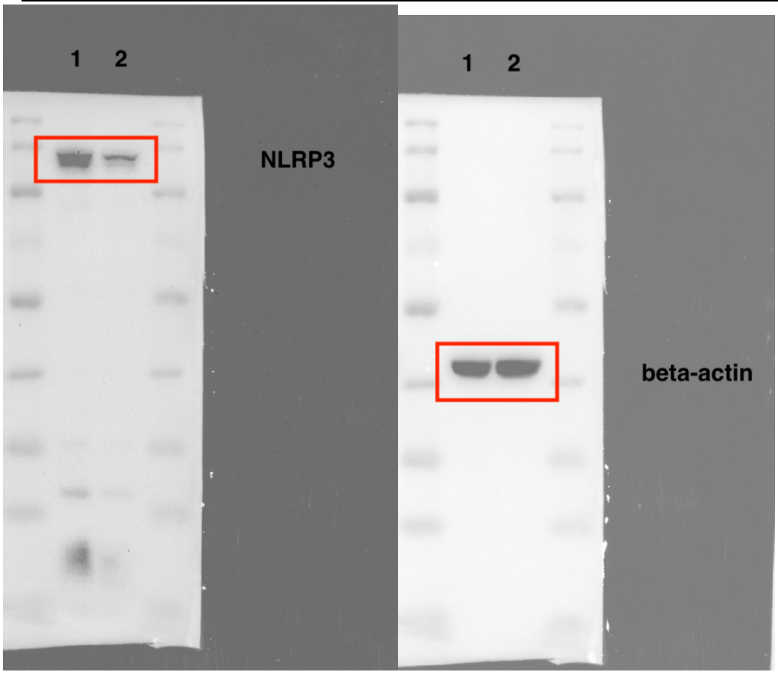

## Figure 5A

| | |
|---|---|
| 1 | PMA_THP-1_scrambled siRNA_Mock |
| 2 | PMA_THP-1_scrambled siRNA_EV-A71_12 hr |
| 3 | PMA_THP-1_scrambled siRNA_EV-A71_24 hr |
| 4 | PMA_THP-1_siTLR3_Mock |
| 5 | PMA_THP-1_siTLR3_12 hr |
| 6 | PMA_THP-1_siTLR3_24 hr |

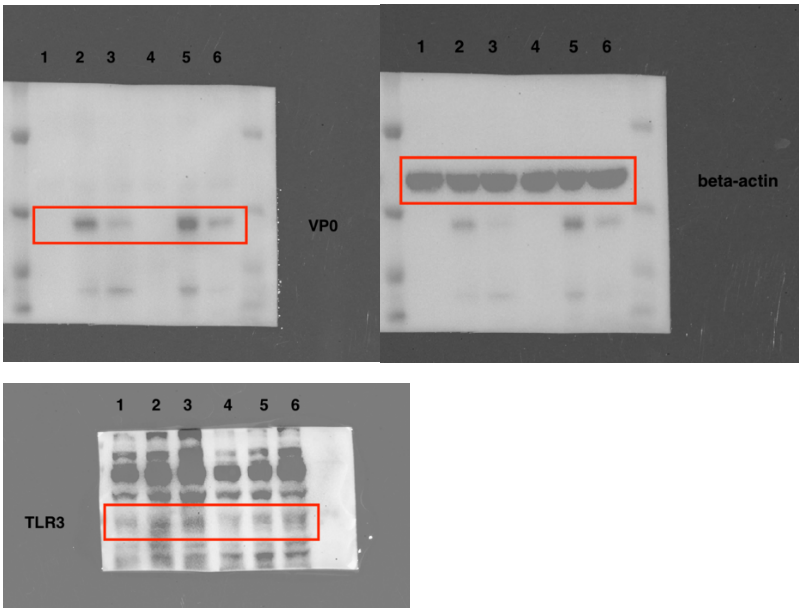


## Figure 5C

| | |
|---|---|
| 1 | PMA_THP-1_scrambled siRNA_Mock |
| 2 | PMA_THP-1_siTLR8_Mock |
| 3 | PMA_THP-1_scrambled siRNA_EV-A71 |
| 4 | PMA_THP-1_ siTLR8_EV-A71 |

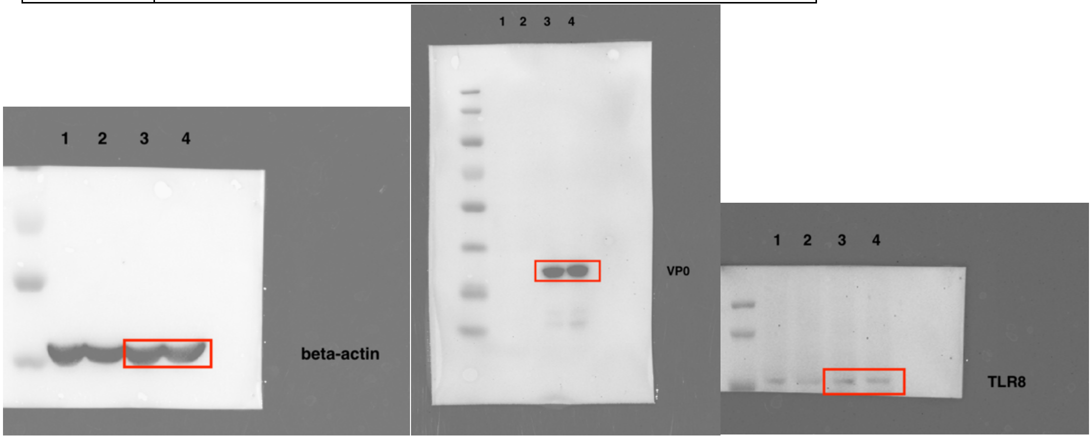

## Figure 5E

| | |
|---|---|
| 1 | PMA_WT THP-1_scrambled siRNA_EV-A71 |
| 2 | PMA_WT THP-1_siTLR3_EV-A71 |
| 3 | PMA_NLRP3 KD THP-1_scrambled siRNA_EV-A71 |
| 4 | PMA_NLRP3 KD THP-1_ siTLR3_EV-A71 |

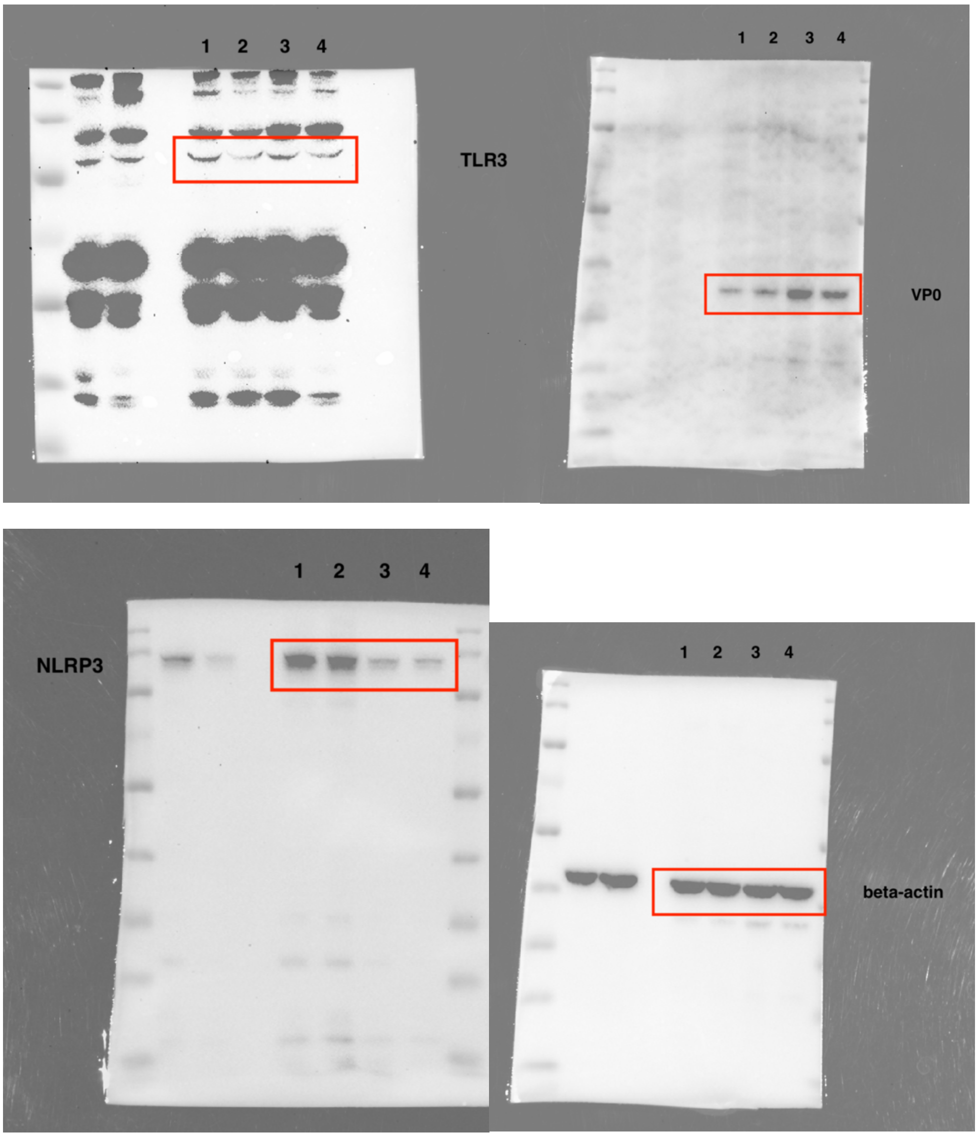

## Figure 6B

| | |
|---|---|
| 1 | PMA_THP-1_scrambled siRNA |
| 2 | PMA_THP-1_siTLR3 |

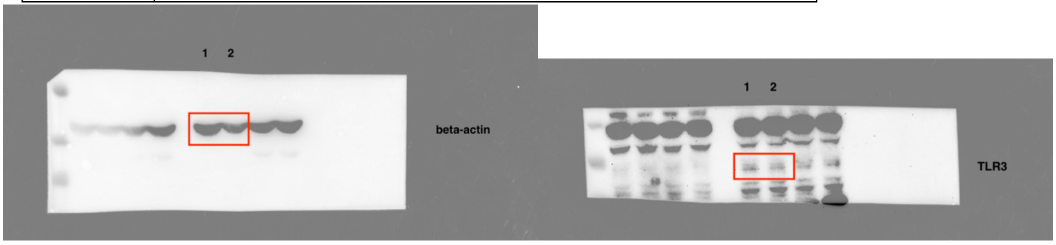


## Figure 6D

| | |
|---|---|
| 1 | PMA_THP-1_shNC |
| 2 | PMA_THP-1_shTLR8 |

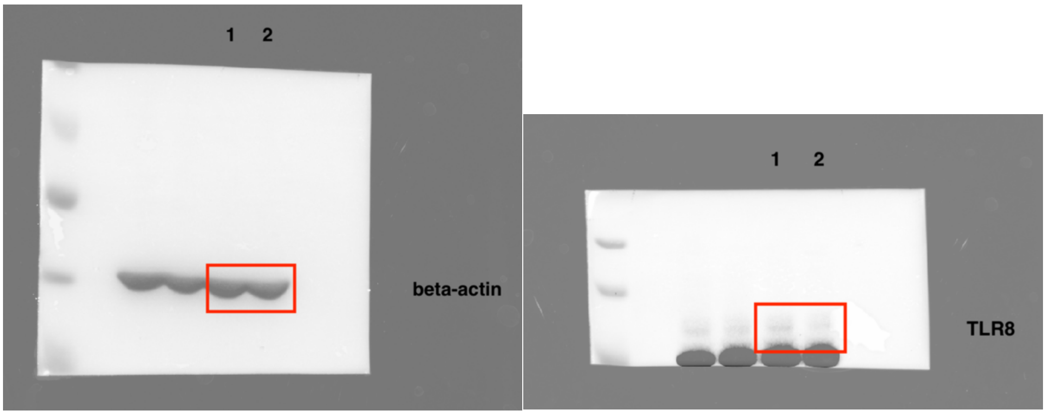


## Figure 6E

| | |
|---|---|
| 1 | PMA_WT THP-1 |
| 2 | PMA_NLRP3 KD THP-1 |

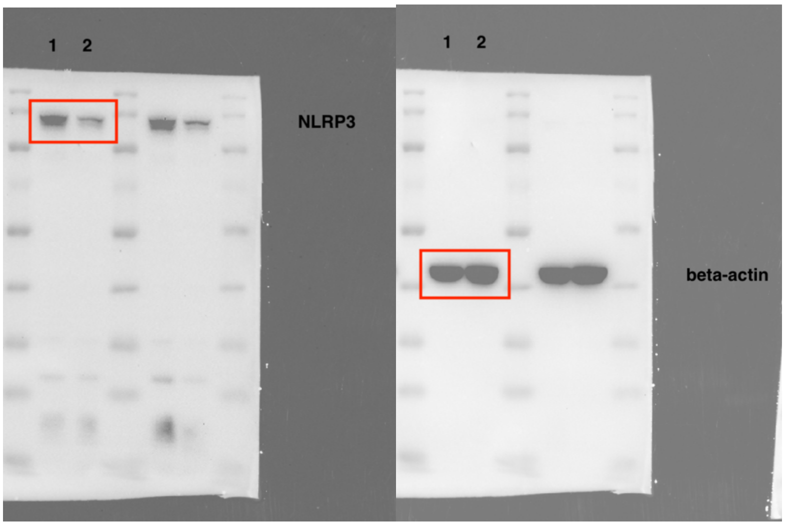

**Figure 6F**

| | |
|---|---|
| 1 | PMA_THP-1_scrambled siRNA |
| 2 | PMA_THP-1_siRIG-I |

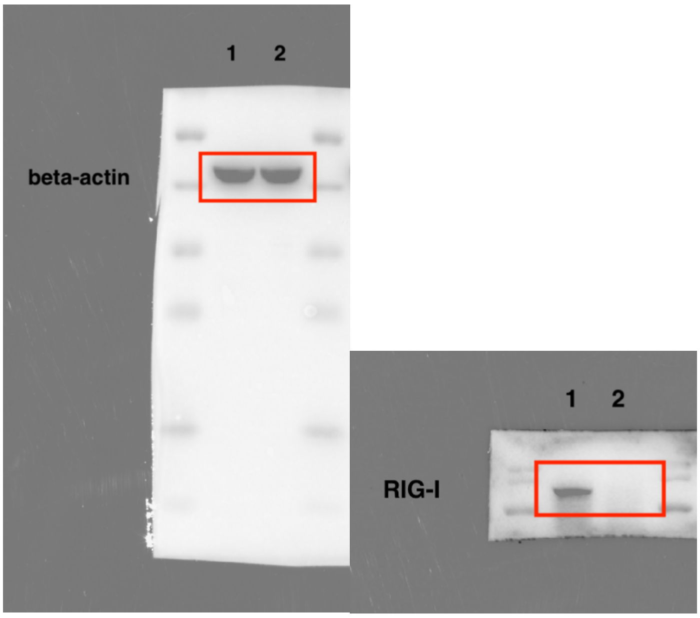

## Figure 7C

| | |
|---|---|
| 1 | PMA_THP-1_untreated_Mock |
| 2 | PMA_THP-1_untreated_EV-A71 |
| 3 | PMA_THP-1_CHX_EV-A71 |

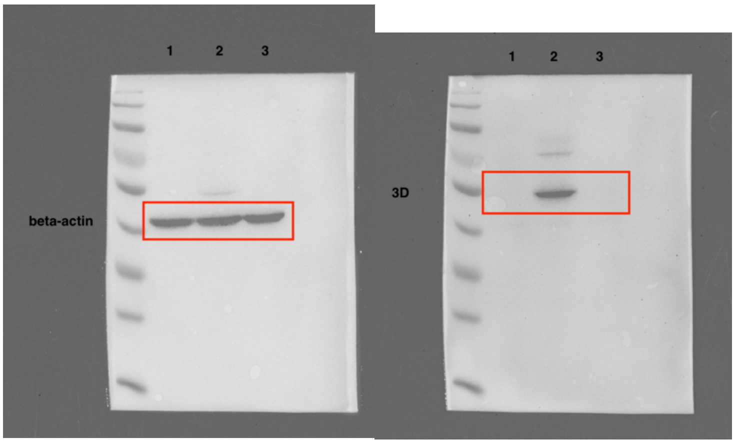


## Figure 7E

| | |
|---|---|
| 1 | PMA_THP-1_untreated |
| 2 | PMA_THP-1_LF2K |
| 3 | PMA_THP-1_LF2K_RD RNA |
| 4 | PMA_THP-1_LF2K_0.5ug EV-A71 vRNA |
| 5 | PMA_THP-1_LF2K_1ug EV-A71 vRNA |
| 6 | PMA_THP-1_LF2K_2ug EV-A71 vRNA |

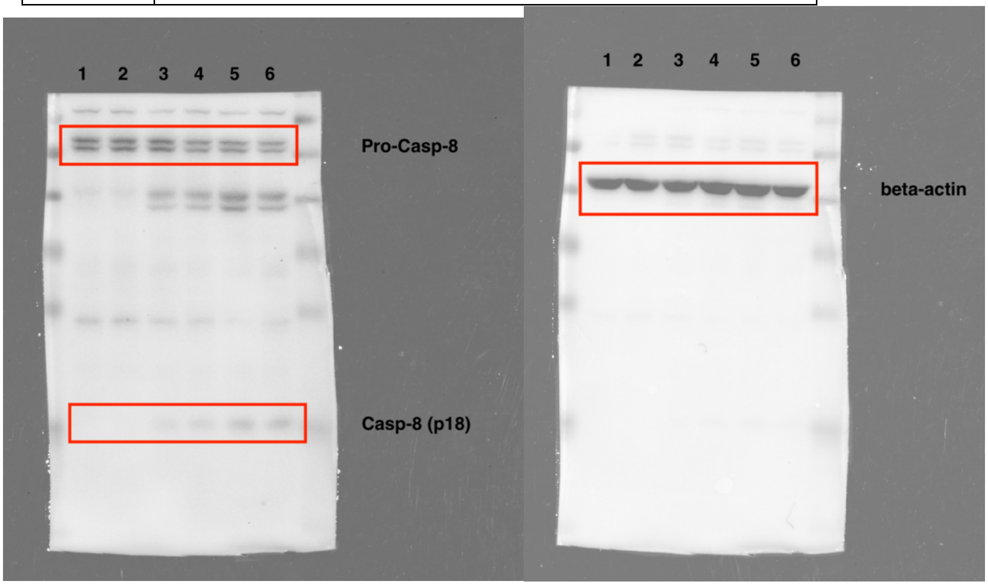

## Supplementary Figure 1B

| | |
|---|---|
| 1 | THP-1_Mock |
| 2 | THP-1_EV-A71_6 hr |
| 3 | THP-1_EV-A71_12 hr |
| 4 | THP-1_EV-A71_24 hr |
| 5 | THP-1_EV-A71_36 hr |
| 6 | THP-1_EV-A71_48 hr |
| 7 | PMA_THP-1_Mock |
| 8 | PMA_THP-1_EV-A71_6 hr |
| 9 | PMA_THP-1_EV-A71_12 hr |
| 10 | PMA_THP-1_EV-A71_24 hr |
| 11 | PMA_THP-1_EV-A71_36 hr |
| 12 | PMA_THP-1_EV-A71_48 hr |

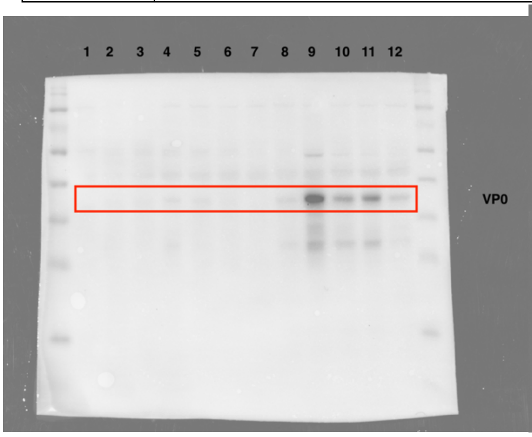


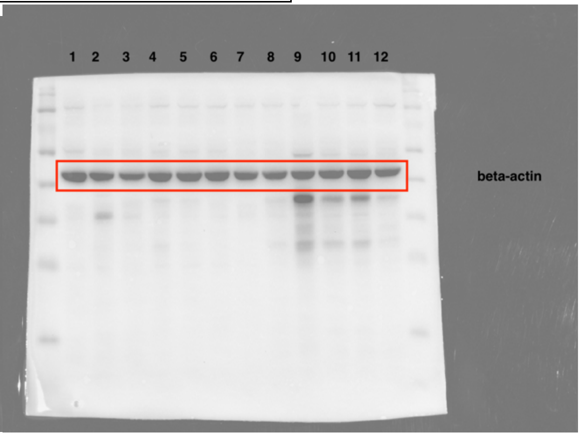


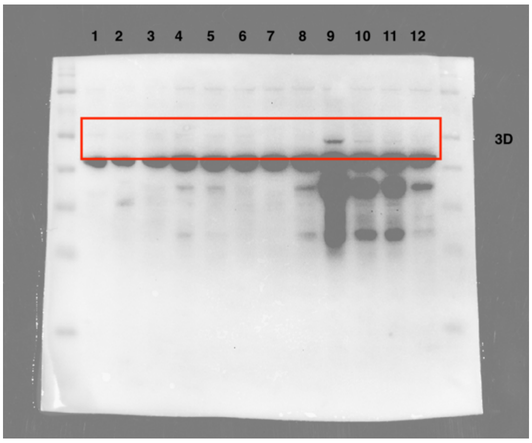

## Supplementary Figure 3

| | |
|---|---|
| 1 | THP-1_untreated |
| 2 | THP-1_LF2K |
| 3 | THP-1_LF2K_poly(A:U) |

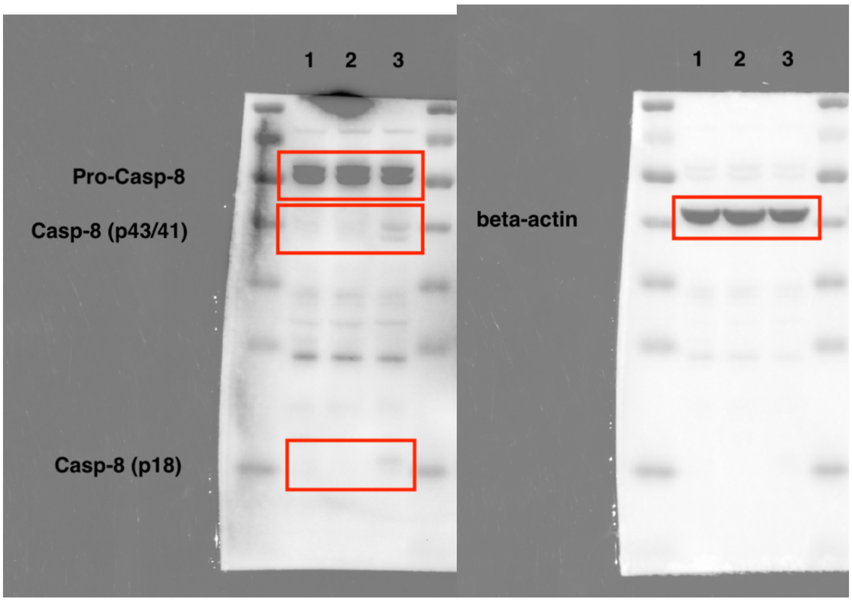

Supplement: Supplementary file 5 — Supplementary Information 5. [file 41598_2022_25458_MOESM5_ESM.pdf]
